# A time-stratified, case–crossover study of heat exposure and perinatal mortality from 16 hospitals in sub-Saharan Africa

**DOI:** 10.1038/s41591-024-03245-7

**Published:** 2024-09-03

**Authors:** Claudia Hanson, Jeroen de Bont, Kristi Sidney Annerstedt, Maria del Rosario Alsina, Federica Nobile, Nathalie Roos, Peter Waiswa, Andrea Pembe, Jean-Paul Dossou, Effie Chipeta, Lenka Benova, Hussein Kidanto, Cherie Part, Massimo Stafoggia, Veronique Filippi, Petter Ljungman

**Affiliations:** 1 Department of Global Public Health, Karolinska Institutet, Stockholm, Sweden; 2 London School of Hygiene and Tropical Medicine, London, UK; 3 Centre of Excellence for Women and Child Health, Aga Khan University, Nairobi, Kenya; 4 Institute of Environmental Medicine, Karolinska Institutet, Stockholm, Sweden; 5 Department of Epidemiology, Lazio Region Health Service/ASL Roma 1, Rome, Italy; 6 Clinical Epidemiology Division, Department of Medicine, Karolinska Institutet, Stockholm, Sweden; 7 Makerere University, Kampala, Uganda; 8 Department of Obstetrics and Gynaecology, Muhimbili University of Health and Allied Sciences, Dar Es Salaam, Tanzania; 9 Centre de Recherche en Reproduction Humaine et en Démographie (CERRHUD), Cotonou, Benin; 10 Centre for Reproductive Health, Kamuzu University of Health Science, Blantyre, Malawi; 11 Department of Public Health, Institute of Tropical Medicine, Antwerp, Belgium; 12 Department of Cardiology, Danderyd Hospital, Danderyd, Sweden

**Keywords:** Epidemiology, Risk factors

## Abstract

Growing evidence suggests that extreme heat events affect both pregnant women and their infants, but few studies are available from sub-Saharan Africa. Using data from 138,015 singleton births in 16 hospitals in Benin, Malawi, Tanzania and Uganda, we investigated the association between extreme heat and early perinatal deaths, including antepartum and intrapartum stillbirths, and deaths within 24 h after birth using a time-stratified case–crossover design. We observed an association between an increase from the 75th to the 99th percentile in mean temperature 1 week (lag 0–6 d) before childbirth and perinatal mortality (odds ratio (OR) = 1.34 (95% confidence interval (CI) 1.01–1.78)). The estimates for stillbirths were similarly positive, but CIs included unity: OR = 1.29 (95% CI 0.95–1.77) for all stillbirths, OR = 1.18 (95% CI 0.71–1.95) for antepartum stillbirths and OR = 1.64 (95% CI 0.74–3.63) for intrapartum stillbirths. The cumulative exposure–response curve suggested that the steepest slopes for heat for intrapartum stillbirths and associations were stronger during the hottest seasons. We conclude that short-term heat exposure may increase mortality risks, particularly for intrapartum stillbirths, raising the importance of improved intrapartum care.

## Abstract (web summary)

Data collected from 138,015 hospital-based singleton births in four sub-Saharan African countries revealed an association between heat exposure in the week leading up to the birth and perinatal mortality.

## Main

Globally, every year 1.9 million infants are stillborn—that is, born dead^1^. There are an additional 2.3 million newborn deaths: these are babies who are born alive but die within the first 28 d of life—the neonatal period^1^. Intrapartum stillbirths, babies dying during labor, comprise roughly 50% of the stillbirth burden in sub-Saharan Africa. Reducing the high burden of stillbirth and neonatal mortality is a main objective of the UN Sustainable Development Goals and the World Health Organization’s Every Newborn Action Plan^2,3^. Although, in recent years, some progress has been seen in perinatal health and survival, hard-won gains could be further hampered by the effects of climate change^4^.

Temperatures are rising globally^5^. In Africa, average rates of warming increased from +0.2 °C per decade between 1961 and 1990 to +0.3 °C per decade during the 1991–2022 period^6^. Emerging evidence suggests that high ambient air temperature is associated with an increased risk of stillbirth^7–12^.

**Fig. 1:**
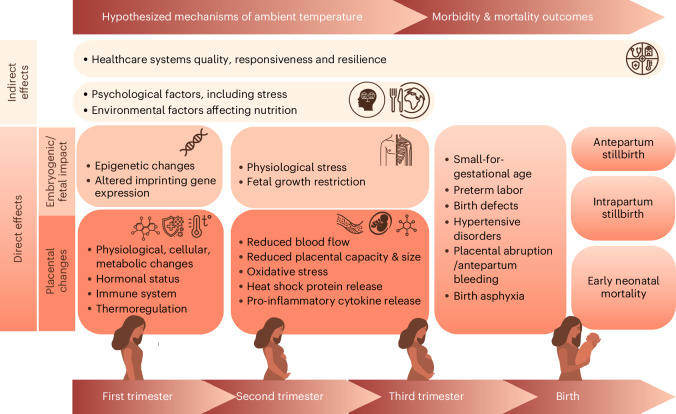
Hypothesized physiological, environmental and health system pathways of the effect of extreme heat on perinatal outcomes. Direct effects of heat may include placental changes and embryogenic/fetal impacts. These may lead to epigenetic changes, imprinting and other molecular effects. In the second and third trimester, these changes may lead to physiological effects, such as fetal growth restriction, resulting in stillbirth and neonatal deaths. In addition, indirect impacts may have an effect through health system responsiveness or through psychological factors, including stress and nutrition.

Scholars have hypothesized pathophysiological pathways, mainly informed by animal studies, broadly categorizing (1) indirect and (2) direct pathways (Fig. 1). Indirect pathways include macro-level factors, such as the negative effects of heat on health system responsiveness due to healthcare workers’ exhaustion or on agriculture affecting nutrition. Direct pathways include embryogenetic and fetal impacts as well as placental changes^11^. Physiological adaptation to high temperatures involves cellular changes and altered hormonal and immune systems that generate placental changes. This might increase the risk of hypertensive disorders^9,13,14^ and placental abruption^15,16^ or trigger contractions^10,17^ that directly affect survival of the fetus. Heat can lead to epigenetic changes or altered imprinting that could result in birth defects^18^, a known risk factor for stillbirth and neonatal death^19^.

Mechanisms at birth may include overwhelmed thermoregulation, which may reduce central blood flow, resulting in placental hypoperfusion^17,20^, dehydration, hypovolemia and other physiological effects^17^. Reduced placental capacity, oxidative stress and heat shock proteins may trigger preterm birth—that is, birth before 37 weeks of gestational age^7,9,21^. Preterm birth is a major risk factor for stillbirth and neonatal mortality^22^. Furthermore, heat stress may impair placental development and spiral artery remodeling and increase the risk of hypertensive disorders, another key risk factor for stillbirths^9,13,14^. Placental abruption has also been associated with ambient temperature, directly affecting survival of the fetus^15,16^.

Epidemiological studies conducted in high-income countries suggest that the risk of stillbirth is due to the acute effect of ambient temperature during the week before birth^23,24^. It is important to note that the underlying and immediate causes of stillbirth differ between settings, which is why the evidence from high-income countries can neither be generalized to sub-Saharan Africa nor fully inform the development of interventions for mitigation. In high-income countries, most stillbirths occur in the antepartum period—that is, before labor starts^23–25^. Causes of antepartum stillbirths are often multifactorial, and no single underlying condition or cause may explain the death^26^. In contrast, intrapartum stillbirths—the predominant type of stillbirth in sub-Saharan Africa—are more often caused by direct obstetric complications, such as prolonged labor or acute fetal distress. However, only two studies, both with a very limited number of intrapartum stillbirths and both from high-income countries, have evaluated the effect of heat by timing or causes of such deaths. The findings from these two studies were inconclusive^27,28^. Thus, there is insufficient evidence on the effects of heat on stillbirths as well as deaths immediately after birth specifically addressing the sub-Saharan African context^7,11^.

In summary, current understanding of the impacts of extreme heat on perinatal mortality is insufficient^7,11^, and disaggregating the effects of heat by time of death is crucial for designing specific public health interventions. For instance, if women and their infants are most vulnerable during birth, infrastructural and operational changes in childbirth facilities will need to be prioritized to address the increased risk^11^.

Within the context of (1) rapidly rising temperatures in Africa, (2) extremely high burden of perinatal mortality in sub-Saharan Africa and (3) lack of studies to investigate the effect of extreme heat on intrapartum stillbirths, our study investigated the association between heat exposure in the final week of pregnancy and perinatal deaths, disaggregated by timing of death (before and during labor). We combined ambient temperature data with a large clinical dataset including births from 16 hospitals in Benin, Malawi, Tanzania and Uganda, and we performed case–crossover analyses disaggregating associations between heat exposure and perinatal mortality and antepartum and intrapartum stillbirths.

## Results

### Population characteristics

This study included 138,015 singleton births from four sub-Saharan African countries between 1 July 2021 and 31 December 2023 (Table 1 and Supplementary Fig. 1). Perinatal deaths included all stillbirths and neonatal deaths occurring during the first 24 h of life (5,542; 4.0%). Stillbirths constituted 4,886 (3.5%) of all births, of which 2,734 (56.0%) were classified as antepartum and 2,152 (44.0%) as intrapartum, based on skin appearance. The largest sample size of births was obtained from Malawi (accounting for 36.6% of the total sample), whereas Tanzania had the lowest sample size (17.3%). Benin had the highest rate of stillbirths and perinatal deaths, accounting for 7.2% and 8.0% of all births, respectively. Benin and Malawi showed the highest and lowest weekly mean temperature before birth of 26.9 °C and 20.1 °C, respectively (Table 1 and Supplementary Fig. 2). Tanzania recorded the highest average maximum temperature of 30.2 °C, and Benin had the highest average minimum temperature of 25.0 °C.

**Table 1:** Childbirth population (number of births) characteristics of 16 hospitals of Benin, Malawi, Tanzania and Uganda (July 2021 to December 2023)

	Benin	Malawi	Tanzania	Uganda
	(*n* = 26,390)	(*n* = 50,491)	(*n* = 23,811)	(*n* = 37,323)
	*n*/(%)			
Outcomes				
Stillbirth	1,893 (7.2)	944 (1.9)	369 (1.5)	1,680 (4.5)
Antepartum stillbirth	1,101 (4.2)	469 (0.9)	159 (0.7)	1,005 (2.7)
Intrapartum stillbirth	792 (3.0)	475 (0.9)	210 (0.9)	675 (1.8)
Perinatal deaths	2,110 (8.0)	1,285 (2.5)	469 (2.0)	2,016 (5.4)
Population characteristics				
Gestational age (mean, s.d.)	38.2 (2.4)	37.8 (1.6)	38.7 (2.2)	38.4 (2.1)
Maternal age (≥35 years)	3,943 (14.9)	4,424 (8.8)	3,792 (15.9)	3,340 (9.0)
Referred to hospital during childbirth	14,191 (53.8)	4,629 (9.2)	1,133 (4.8)	5,246 (14.2)
HIV positive	353 (1.6)	1,658 (3.8)	730 (3.1)	1,191 (3.4)
Hypertensive disorders	5,032 (19.1)	877 (1.7)	1,129 (4.7)	1,815 (4.9)
Two or more pregnancies	19,100 (72.4)	27,542 (54.6)	15,325 (64.4)	24,760 (66.4)
Sex (female)	13,866 (52.6)	26,033 (51.6)	12,022 (50.5)	18,595 (49.8)
Preterm births	4,432 (17.0)	5,284 (10.6)	3,197 (13.5)	3,935 (11.1)
Low birth weight (<2,500 g)	4,982 (19.0)	6,262 (12.4)	2,652 (11.1)	3,415 (9.2)
Prolonged/obstructed labor	1,617 (6.1)	4,210 (8.3)	2,390 (10.0)	2,540 (6.8)
Delivery mode				
Spontaneous vaginal	13,578 (51.5)	41,171 (81.5)	16,887 (70.9)	26,852 (72.0)
Cesarean	12,477 (47.3)	8,561 (17.0)	6,758 (28.4)	10,347 (27.7)
Assisted vaginal birth	332 (1.3)	759 (1.5)	165 (0.7)	120 (0.3)
Antepartum hemorrhage	740 (2.8)	350 (0.7)	170 (0.7)	663 (1.8)
Temperature indicators^a^				
Mean temperature, °C (s.d.)	26.9 (1.2)	20.1 (2.9)	24.8 (1.6)	22.1 (0.8)
Minimum temperature, °C (s.d.)	25.0 (1.1)	15.3 (3.1)	20.3 (2.0)	18.8 (0.8)
Maximum temperature, °C (s.d.)	29.8 (1.8)	25.2 (3.0)	30.2 (2.2)	25.9 (1.4)

Values are mean (s.d.) for continuous normal distributed variables, median (interquartile range) for continuous non-normal distributed variables and percentage for categorical variables.

^a^Mean values, week before birth (lag 0–6 d).

### Association between temperature and perinatal mortality

**Fig. 2:**
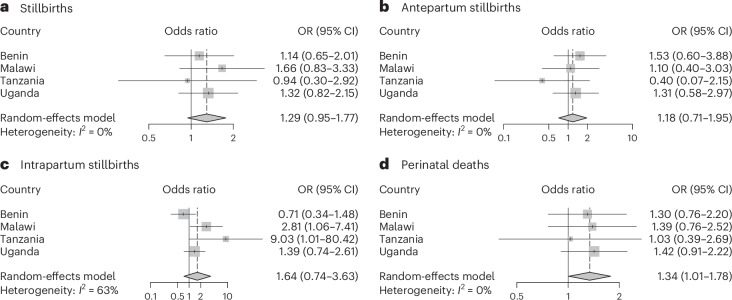
Country-specific and pooled estimates showing the associations of an increase of temperature the week before birth (lag 0–6 d) from the 75th to the 99th percentile and perinatal mortality of 16 hospitals in Benin, Malawi, Tanzania and Uganda. This figure depicts the country-specific and pooled estimates presented as ORs and 95% CIs (mean ± 1.96 × s.e.m.) obtained from conditional logistic regression models. The estimates show the association of an increase in temperature (from the 75th to the 99th percentile) the week before birth (lag 0–6 d) on stillbirths (*n* = 4,886) (**a**), antepartum stillbirths (*n* = 2,152) (**b**), intrapartum stillbirths (*n* = 2,734) (**c**) and perinatal deaths (*n* = 5,880) (**d**). In the case–crossover design, each case serves as its own control. We defined control days as the same day of the week within the same month as childbirth. The data were obtained from 16 hospitals in four different countries, including Benin (four hospitals), Malawi (four hospitals), Tanzania (four hospitals) and Uganda (four hospitals). The vertical dashed line represents the value of the OR obtained from the meta-analyses, and the size of the square corresponds to the weight contribution to the meta-analyses. Estimates with smaller CIs had larger squares and contributed with more weight to the meta-analyses.

**Extended Data Fig. 1:**
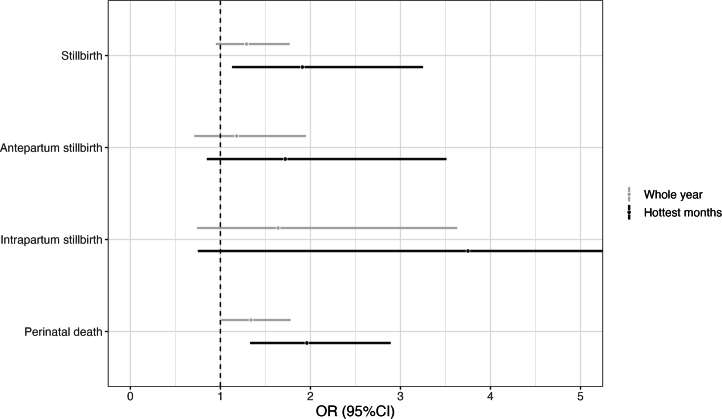
Associations between mean temperature the week before birth (lag 0–6) and perinatal mortality during the 6 hottest consecutive months. Sensitivity Analysis: The figure depicts the pooled estimates for the whole year (grey line, main analysis) and during the 6 hottest consecutive months (black line). The estimates are presented as odds ratio (OR) and 95% confidence intervals obtained from conditional logistic regression models. The estimates show the association of an increase in temperature (from the 75^th^ to the 99^th^ percentile) the week before birth (lag 0–6) on stillbirths, antepartum stillbirths, intrapartum stillbirths, and perinatal deaths.

We observed an association (odds ratio (OR) = 1.34, 95% confidence interval (CI): 1.01–1.78, *I*^2^ = 0%) between heat exposure and perinatal mortality with an increase from the 75th to the 99th percentile in mean temperature 1 week (lag 0–6 d) before childbirth (Fig. 2 and Supplementary Table 2). We found similar positive risk estimates for stillbirths with CIs including unity: OR = 1.29 (95% CI 0.95–1.77, *I*^2^ = 0%) for all stillbirths; OR = 1.18 (95% CI 0.71–1.95, *I*^2^ = 0%) for antepartum stillbirths and OR = 1.64 (95% CI 0.74–3.63, *I*^2^ = 63%) for intrapartum stillbirths. Associations were stronger during the six hottest months of the year with OR = 1.91 (95% CI 1.13–3.25) for stillbirths and OR = 1.96 (95% CI 1.33–2.89) for all perinatal deaths (Extended Data Fig. 1). Tanzania, which had the smallest sample size, demonstrated more unstable effect estimates with wide CIs for antepartum and intrapartum stillbirths. Country-specific associations were otherwise consistent and showed positive associations, with the exception of antepartum stillbirths in Benin, which included one tertiary hospital with very high referral rates.

**Extended Data Fig. 2:**
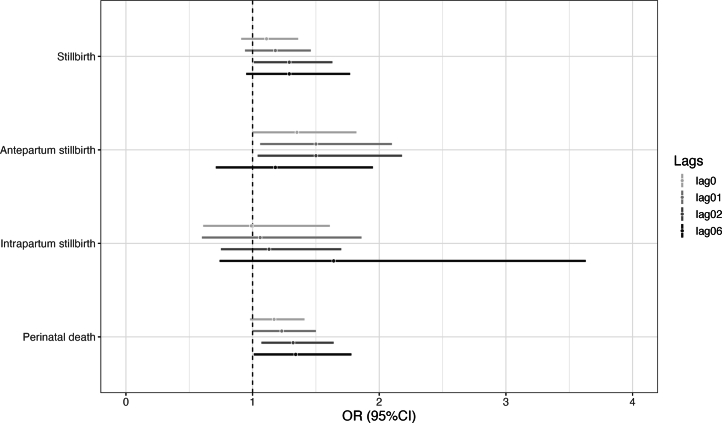
Associations between mean temperature the week before birth (lag 0–6) and perinatal mortality by different lags (0, 0–1, 0–2 and 0–6). Sensitivity analysis: The figure depicts the pooled estimates for increasing numbers of lags: 0, 0–1, 0–2, and 0–6 (with the last being our main approach). The estimates are presented as odds ratio (OR) and 95% confidence intervals obtained from conditional logistic regression models. The estimates show the association of an increase in temperature (from the 75^th^ to the 99^th^ percentile) the week before birth (lag 0–6) on stillbirths, antepartum stillbirths, intrapartum stillbirths, and perinatal deaths.

**Extended Data Fig. 3:**
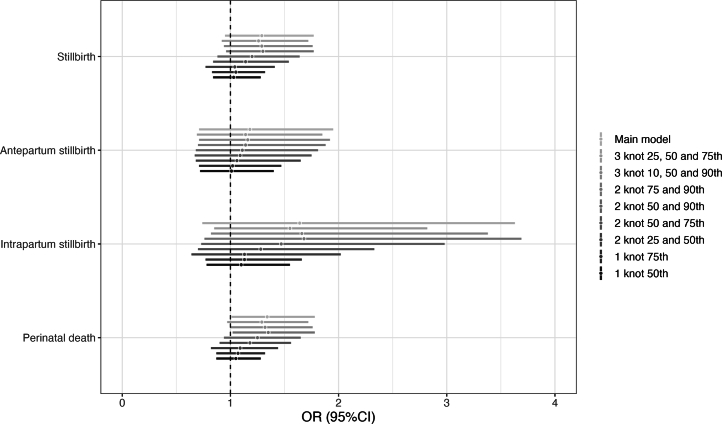
Associations between mean temperature and perinatal mortality by number of knots and their location. Sensitivity analysis: The figure depicts the pooled estimates evaluating heat effect by applying a varying number of knots and adjusting their locations (1 knot at the 50^th^ and 75^th^, 2 knots at the 25^th^ and 50^th^, 2 knots at the 50^th^ and 75^th^, 2 knots at the 50^th^ and 90^th^, 2 knots at the 75^th^ and 90^th^, 3 knots at the 10^th^, 50^th^, and 90^th^, and 3 knots at the 25^th^, 50^th^, and 75^th^). Our main model included 3 knots at the 10^th^, 75^th^ and 90^th^ percentile. The estimates are presented as odds ratio (OR) and 95% confidence intervals obtained from conditional logistic regression models. The estimates show the association of an increase in temperature (from the 75^th^ to the 99^th^ percentile) the week before birth (lag 0–6) on stillbirths, antepartum stillbirths, intrapartum stillbirths, and perinatal deaths.

**Extended Data Fig. 4:**
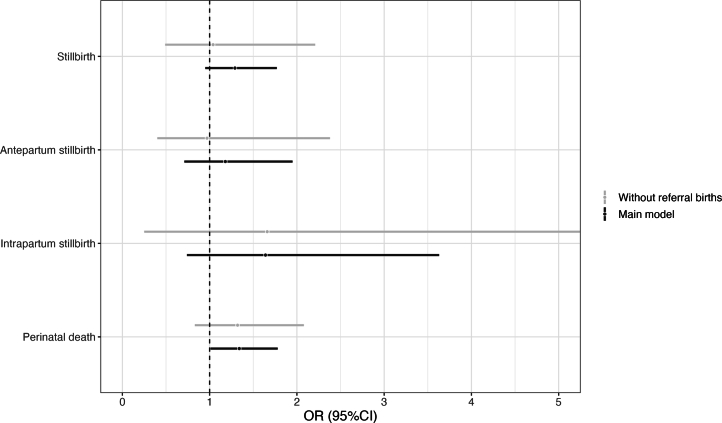
Associations between mean temperature the week before birth (lag 0–6) and perinatal mortality comparing the main exposure model and excluding the referral births. Sensitivity analysis: The figure depicts the pooled estimates from our main analysis (black line) and excluding the referral births (grey line). Women referred as the patients may have travelled much greater distances. This non-referred sub-population may also be closer to the general birthing population The estimates are presented as odds ratio (OR) and 95% confidence intervals obtained from conditional logistic regression models. The estimates show the association of an increase in temperature (from the 75^th^ to the 99^th^ percentile) the week before birth (lag 0–6) on stillbirths, antepartum stillbirths, intrapartum stillbirths, and perinatal deaths.

**Extended Data Fig. 5:**
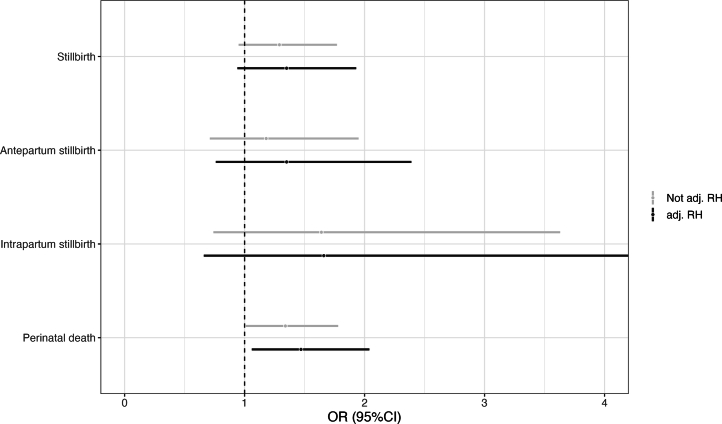
Associations between mean daily temperature the week before birth (lag 0–6) and perinatal mortality adjusted by relative humidity. Sensitivity analysis: The figure depicts the pooled estimates adjusting (black line) and not adjusting (grey line, main approach) for relative humidity. The estimates are presented as odds ratio (OR) and 95% confidence intervals obtained from conditional logistic regression models. The estimates show the association of an increase in temperature (from the 75^th^ to the 99^th^ percentile) the week before birth (lag 0–6) on stillbirths, antepartum stillbirths, intrapartum stillbirths, and perinatal deaths.

**Extended Data Fig. 6:**
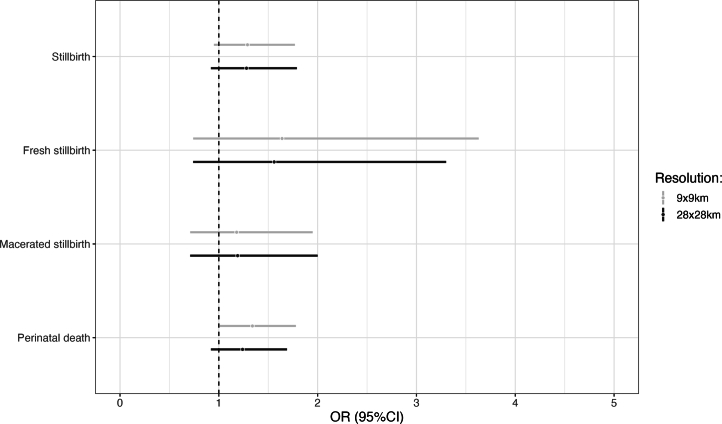
Associations between mean temperature the week before birth (lag 0–6) and perinatal mortality comparing the main exposure model (resolution 9 × 9 × m) and lower resolution model (28 × 28 km). Sensitivity analysis: The figure depicts the pooled estimates comparing the main exposure model at a 9 × 9 km spatial resolution (grey line) and lower 28 × 28 spatial resolution model (black line). The estimates are presented as odds ratio (OR) and 95% confidence intervals obtained from conditional logistic regression models. The estimates show the association of an increase in temperature (from the 75^th^ to the 99^th^ percentile) the week before birth (lag 0–6) on stillbirths, antepartum stillbirths, intrapartum stillbirths, and perinatal deaths.

**Extended Data Fig. 7:**
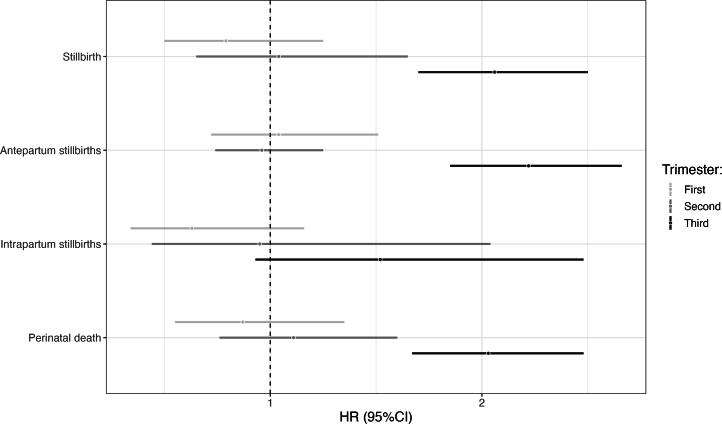
Associations between trimester-specific mean temperatures (99^th^ vs 75^th^ percentile) during pregnancy and perinatal mortality. Sensitivity analyses: The figure depicts the pooled estimates comparing trimester-specific mean temperatures (99^th^ vs 75^th^ percentile) during pregnancy and perinatal mortality. The estimates are presented as hazard ratios (HR) and 95% confidence intervals obtained from a Cox proportional hazard model. Models were adjusted for month of conception, year of conception, sex of child, maternal age and number of pregnancies. The estimates show the association of an increase in mean temperature (from the 75^th^ to the 99^th^ percentile) during the first, second and third trimester on stillbirths, antepartum stillbirths, intrapartum stillbirths, and perinatal deaths.

Exploring the influence of modeling different exposure duration periods during the last week of pregnancy on the associations between temperature and birth outcomes, we observed similar associations between stillbirths and all perinatal deaths with exposure during the last two (lag 0–2) and the last seven (lag 0–6) days before birth (Extended Data Fig. 2). We observed the strongest evidence of an association using lag 0–2 d for antepartum stillbirths (OR = 1.50 (95% CI 1.04–218)) and lag 0–6 d for intrapartum stillbirths. By incorporating two or three knots at higher percentiles, we found results that were consistent with our primary approach (as shown in Extended Data Fig. 3). However, with fewer knots at lower-percentile locations (that is, less flexibility at high temperatures), we were unable to adequately capture the effects of heat. We found very similar associations using maximum and minimum temperatures 1 week before birth (Supplementary Table 2) but slightly stronger associations using minimum temperatures for all stillbirths and maximum temperatures for intrapartum stillbirths. When excluding births of women who were not referred, thus probably more similar to a general birthing population in the countries, results remained largely consistent, except for stillbirths, where the effect estimate was reduced, although the CIs overlapped with those of the main model results (Extended Data Fig. 4). In other sensitivity analyses, we observed similar associations using the 50th percentile as centered values (Supplementary Table 3), and, additionally, the effect estimates of temperature and perinatal mortality did not change after adjusting for relative humidity (Extended Data Fig. 5). Finally, we observed almost identical estimates using the higher (9 km × 9 km) and the lower (28 km × 28 km) resolution temperature models (Extended Data Fig. 6). Finally, we observed that an increase in mean temperature from the 75th to the 99th percentile in the third trimester was associated with a higher hazard ratio for stillbirths and perinatal mortality. No associations were found for the first and second trimesters (Extended Data Fig. 7).

### Exposure–response curve

**Fig. 3:**
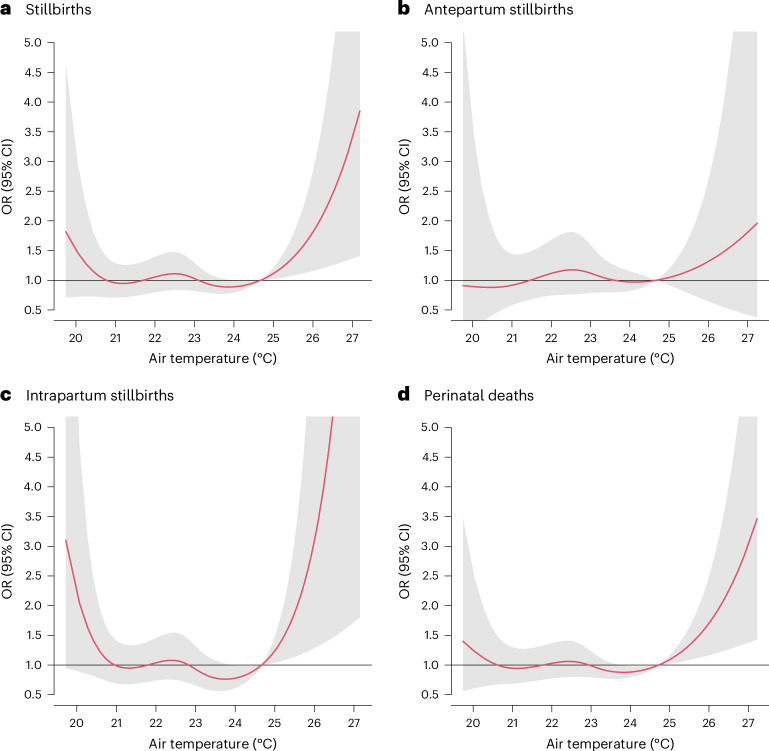
Cumulative exposure–response curve between mean temperature 1 week before childbirth (lag 0–6 d) and perinatal mortality with 95% CIs. The figure depicts the cumulative OR of stillbirths (*n* = 4,886) (**a**), antepartum stillbirths (*n* = 2,152) (**b**), intrapartum stillbirths (*n* = 2,734) (**c**) and perinatal deaths (*n* = 5,880) (**d**) and 95% CIs (mean ± 1.96 × s.e.m.) (gray shadings). In the case–crossover design, each case serves as its own control. We defined control days as the same day of the week within the same month as childbirth. The figure represents the OR of temperature against the cumulative 75th percentile of annual mean temperature. The corresponding 75th percentile temperature values of each country can be found in Supplementary Table 2.

**Extended Data Fig. 8:**
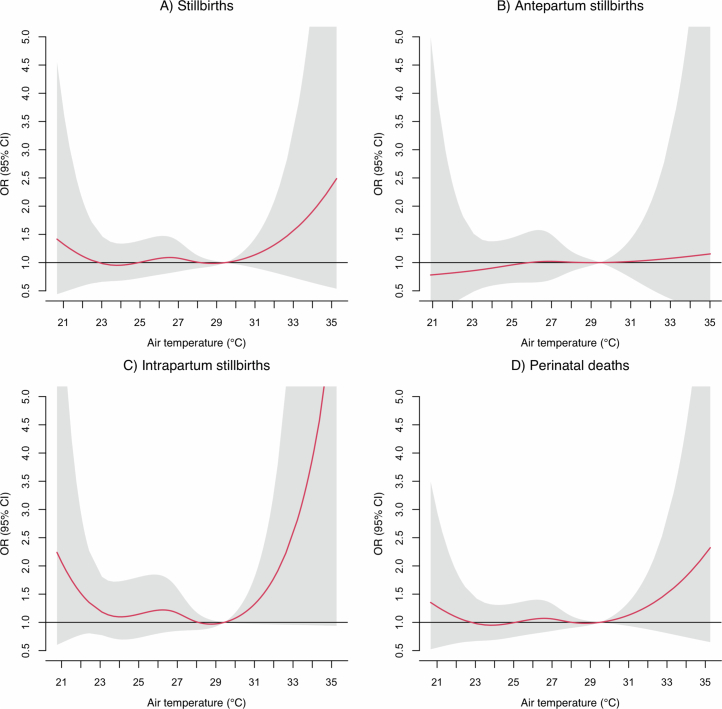
Cumulative exposure-response curve between maximum temperature the week before birth (lag 0–6) and perinatal mortality with 95%Cis. The figure depicts the cumulative odds ratio of (**a**) stillbirths, (**b**) antepartum stillbirths, (**c**) intrapartum stillbirths, and (**d**) perinatal deaths, and 95% CIs (grey shadings). The figure represents the odds ratio of temperature against the cumulative 75^th^ percentile of annual mean maximum temperature. The corresponding 75^th^ temperature values of each country can be found in Supplementary Table 2.

**Extended Data Fig. 9:**
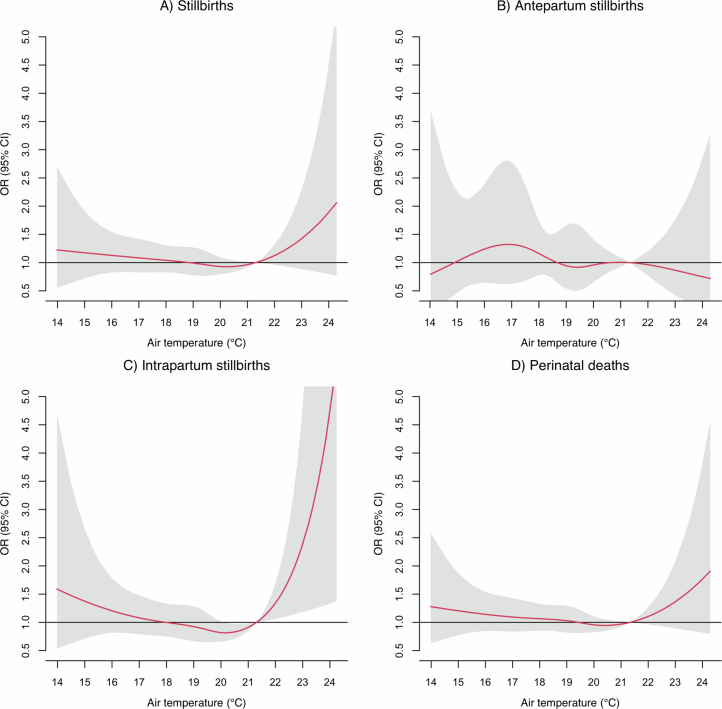
Cumulative exposure-response curve between minimum temperature the week before birth (lag 0–6) and perinatal mortality with 95%Cis. The figure depicts the cumulative odds ratio of (**a**) stillbirths, (**b**) antepartum stillbirths, (**c**) intrapartum stillbirths, and (**d**) perinatal deaths, and 95% CIs (grey shadings). The figure represents the odds ratio of temperature against the cumulative 75th percentile of annual mean minimum temperature. The corresponding 75th temperature values of each country can be found in Supplementary Table 2.

In the meta-regression, we observed a wide U-shaped exposure–response curve between mean temperature and stillbirths and all perinatal deaths, indicating an elevated risk of stillbirths going from the 75th (around 25 °C) percentile until the highest temperatures (Fig. 3). The steepest slope and highest risk were observed for intrapartum stillbirths. Consistent cumulative exposure–response curves were found using maximum and minimum temperatures, indicating that the strongest associations were for intrapartum stillbirths for both extreme sides of temperature (Extended Data Figs. 8 and 9).

### Effect modification

**Extended Data Fig. 10:**
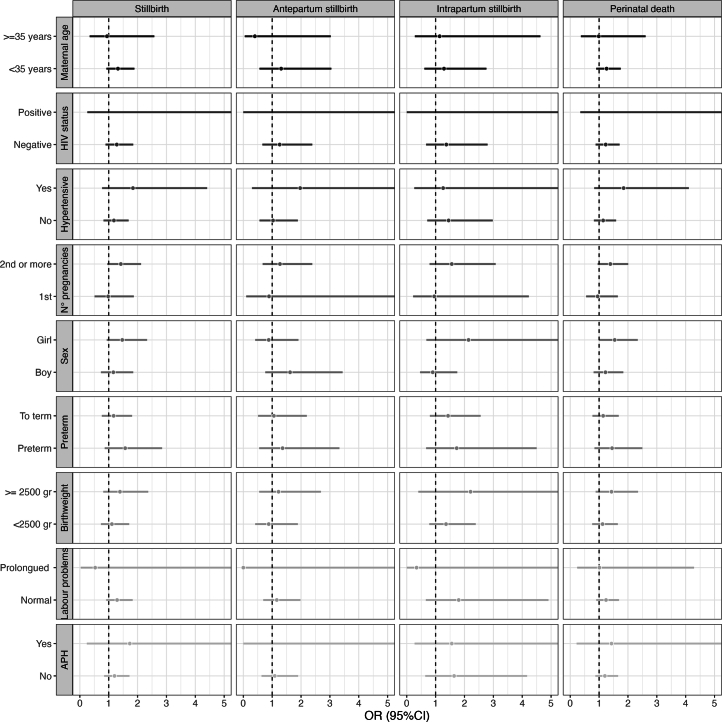
Stratified analyses of effect modifiers in the associations between high temperature the week before birth (99^th^ vs 75^th^ percentile) and perinatal mortality. The figure depicts the stratified pooled estimates by some key effect modifiers that could potentially modify the associations between short-term exposure of ambient air pollution and still births. The key modifiers included maternal age, HIV status, hypertensive disorders, number of pregnancies, sex of the child, preterm births, low birthweight, labour complication and antepartum haemorrhage (APH). The estimates are presented as odds ratio (OR) and 95% confidence intervals obtained from conditional logistic regression models. The estimates show the association of an increase in temperature (from the 75^th^ to the 99^th^ percentile) the week before birth (lag 0–6) on stillbirths, antepartum stillbirths, intrapartum stillbirths, and perinatal deaths.

In effect modification analyses, we found slightly stronger associations between mean temperature and the different stillbirth outcomes in babies of younger women (<35 years) and multiparous women and in preterm births (Extended Data Fig. 10). Furthermore, the effect estimates for HIV-positive women, women with hypertensive disorders and women who gave birth to a preterm baby and extreme heat were higher for stillbirths, intrapartum stillbirths and all perinatal deaths combined but with wide CIs.

## Discussion

Among 138,015 hospital-based singleton births in four sub-Saharan African countries, we observed evidence to support associations between heat exposure in the week leading up to the birth and perinatal mortality. An increase in mean temperature from the 75th to the 99th percentile was associated with a 34% increased risk of perinatal mortality. Effect estimates for stillbirths were in the same direction although did not reach statistical significance but suggested the steepest increase for intrapartum stillbirth. Stronger associations were observed for stillbirths and perinatal mortality during the six hottest months of the year, further supporting these results. This study had a high number (44%) of stillbirths that occurred during labor. Our study adds evidence to a recent narrative review reporting positive associations between heat and stillbirth in 19 of 20 published studies^11^ and previous systematic reviews^7,29^, increasing the number of studies reporting on the effect in sub-Saharan Africa to a total of four studies.

The present study took place in countries with high perinatal mortality. We included larger district or referral hospitals that bear a larger share of births with obstetric complications although with wide variations among the countries. The hospitals in Benin had the highest share of cases that were referred and had obstetric complications, thus a high-risk case mix. The hospitals of Malawi, Tanzania and Uganda had lower complication patterns, suggesting a more unselected case mix. A comparative study using population-based data described that about 30% of all live births take place in hospitals in Benin, 33% in Malawi, 32% in Tanzania and 37% in Uganda^30^, supporting the relevance of our findings despite the distinct case mix and higher-risk birthing population.

Comparing our effects of ambient temperature on stillbirth mortality with other estimates is challenging as studies are heterogeneous in terms of exposure metric, unit change and time period of effect. Generally, evidence is stronger for an acute effect of heat (that is, in the last weeks or trimester of pregnancy)^31–36^ than for a delayed effect occurring in the first or second trimester^28,36^. Few studies have evaluated associations between temperature in the week before birth and stillbirths, as we did. A recent systematic review and meta-analysis^7^ included two studies from high-income countries using the same analysis method (lag 0–6 d). This study reported an OR = 1.24 (95% CI 1.12–1.36) increased risk of stillbirth for extreme heat exposure in the week before birth, which is consistent with our estimate for all stillbirths^7^.

The higher susceptibility toward heat in the third trimester and the last few weeks of pregnancy^23,28,37,38^ motivated us to specifically examine the last week before birth. The time-stratified case–crossover design adjusts for individual-level confounders that change slowly over time (such as maternal age, wealth, gestational age, education, lifestyle and socioeconomic conditions) as well as potential confounding by season and day of the week. This design is particularly efficient at capturing triggered health effects from short-term exposures and on outcomes that are temporally well defined. This might explain why we found the strongest effect on intrapartum stillbirth where we have the highest confidence of timing of death and, thus, least risk of exposure misclassification. The stronger effect may also reflect indirect effects of heat impacts on quality of healthcare delivery. However, our design did not allow analysis of longer-term windows of susceptibility to ambient temperature (for example, multiple weeks) as only short lags can be used with confidence in case–crossover analyses^21^. Thus, our methodology was not suited to capture the effect of heat in early pregnancy, such as on epigenetic changes, imprinting and direct placental changes, nor on prenatal effects of long-term exposure to temperature (Fig. 1)^39^. In our sensitivity analyses, we observed that longer-term exposure during the third trimester was associated with perinatal mortality, supporting the focus on the exposure during the last part of pregnancy. However, the long-term results should be interpreted with caution because we lacked key confounders, such as smoking, wealth and education, among others, that are important to adjust for when using analytical methods, such as time-to-event analyses of long-term exposure. Additionally, because we had no information on the residential addresses of the pregnant women, considering longer periods of exposure will introduce a high risk of exposure misclassification due to a lack of spatial contrast in exposure. Despite this, it is important to note that vulnerabilities that accumulate at birth—for example, placenta insufficiency caused by heat stress in early pregnancy—may be worsened by acute heat stress causing perinatal mortality.

We observed stronger estimates for associations between extreme temperature and intrapartum stillbirths compared to antepartum stillbirths or perinatal deaths. To our knowledge, only two previous studies, both from high-income countries, have attempted to report heat effects by timing of fetal death, and findings were inconclusive^27,28^. Ha et al.^28^ reported similar effect sizes of extreme heat on antepartum versus intrapartum stillbirths across 12 clinical centers across the United States. Auger et al.^27^ examined the effect of heat by cause of stillbirth in Canada and found some evidence that heat was more strongly associated with an ‘undetermined’ cause of death, which is more commonly seen for antepartum stillbirths^27^. However, this study also reported weak evidence of an association between extreme heat and maternal complications, such as premature rupture of membranes—a complication leading more often to intrapartum stillbirth^27^. Furthermore, in a study conducted in the United States, the risk of stillbirth associated with the effect of elevated temperature was higher among women with placental abruption^16^. In our stratified analysis, we found no evidence of an association between heat and women with antepartum hemorrhage, a possible sign and symptom of placental abruption. In addition, we also found no evidence of an association between heat and prolonged labor, another frequent cause of intrapartum stillbirths, thus not supporting the hypothesis that physical exhaustion and prolonged labor may be an important pathway. However, it is important to note that obstetric risk factors are interrelated. For example, hypertensive disorders, depending on severity, may lead to partial or full placental abruption, which may cause either an antepartum or an intrapartum stillbirth. Although recognizing the large uncertainty reflected by the wide CIs, we observed a tendency for higher risks related to heat on overall perinatal deaths, stillbirth and antepartum stillbirth in women with hypertensive disorders, supporting previous evidence of a potential causal mechanism^9,13,14^. However, it is important to note that our results consider heat effects only in late pregnancy. Some previous studies supported the hypothesis that heat exposure in very early pregnancy increases the risk of preeclampsia, which may then increase the risk of perinatal death (Fig. 1)^9,13,14^. Our results point to an additional effect in the last week of pregnancy, further increasing the fetal vulnerability to heat exposure. Results disaggregating the effect by HIV status were inconclusive in contrast with a study from Botswana that reported increased vulnerability to seasonal temperature changes in HIV-positive women^40^.

The main methodological strength of our study was the inclusion of a large number of stillbirths and perinatal deaths from an overall population of 138,015 singleton births. The observations were collected from diverse settings, including private and public facilities and community, district and referral hospitals from four countries in two different regions of sub-Saharan Africa with very different backgrounds. We used a prospective design to analyze the associations between extreme heat exposure and stillbirth and very early neonatal mortality from sub-Saharan Africa. The prospective nature reduces the misclassification error in determining the outcome and made it possible to disaggregate antepartum and intrapartum stillbirths and to expand the analysis to perinatal mortality. Although the data collection started in 2021 during the coronavirus disease 2019 (COVID-19) pandemic, we think that the pandemic had no major impact. First, our data showed no change in the average number of births per hospitals—a pattern also reported by others^41^. Second, there were limited changes in care in our study hospitals, as reported elsewhere^42^.

This study conducted in sub-Saharan Africa disaggregated antepartum and intrapartum stillbirths and perinatal mortality to examine the effect of extreme heat. Intrapartum stillbirths have become rare in most high-income settings because of appropriate obstetric management, but they contribute to almost half of all stillbirths in sub-Saharan Africa, which is why we think that our analysis is of particular relevance^25^.

However, our study also had limitations. First, this is a hospital-based study and, thus, includes a selected birthing population with a relatively high proportion of childbirth complications. In Benin and Uganda, 53.8% and 14.5% of women were referred, respectively, suggesting a very high risk of adverse events. Other analyses of this sample highlight the association between obstetric risk factors and perinatal mortality^43^. Considering that our study includes births taking place only in hospitals, but not in lower-level health centers or at home, we caution against generalizing our findings to wider populations. Our analysis is representative of only about 30–40% of all births that take place in hospitals, and studies from sub-Saharan Africa indicate that poorer women living in remote and rural areas, as well as those women with many children, are underrepresented in hospital birthing populations^30,44^. However, such women may be more vulnerable to heat stress.

Although our data collection approach aimed to ensure quality reporting of clinical risk factors, potential low reporting of factors, such as milder hypertensive disorders, must be taken into consideration. However, these limitations are not likely to confound our results because the case–crossover design analyzes associations within individual comparisons, but they might overlook differences in susceptibilities in subpopulations. One of the assumptions of the individual-level case–crossover design is invariance of the individual-level baseline risk in the referent window (the month of birth, in our example). This is usually considered a reasonable assumption with outcomes such as mortality or hospital admissions, but it might not hold entirely for stillbirths, whose risk is expected to increase in the last weeks of gestation. Although we expect that the bi-directional approach of control selection would partially minimize this risk, we cannot entirely rule out residual bias in our effect estimates^45^. Furthermore, we did not know the exact time of death of the antepartum births, which might have introduced misclassification bias. The hospital-based data collection meant that we must assume that the temperature in the hospital was similar to the women’s home. Catchment areas of hospitals typically include a radius of 10–30 km as we described, for example, in Tanzania^46^. The granularity of the Copernicus Climate Data used included both 28-km and 9-km grids that gave similar results (https://cds.climate.copernicus.eu/), and, thus, we think that the potential bias introduced by using the hospital location instead of the mothersʼ place of residence is acceptable^47^. This is further supported by the fact that short-term temperature changes have higher temporal variability compared to spatial variability. In other words, relative changes in temperature will be similar across rather large areas. Moreover, mean temperature has typically been assumed to be the appropriate temperature metric relevant to health effects; we tested that assumption in sensitivity analyses and observed some evidence to indicate stronger effects using the maximum temperature, similar to some previous studies^31,33^.

Our findings are in line with previous studies and lead to an increased understanding of the effect of extreme heat on perinatal mortality; however, more research is required to understand the pathophysiology behind effects of extreme heat on intrapartum stillbirths versus antepartum stillbirths. Large datasets with clinical, physiological, socio-demographic and behavioral data are required for this type of analysis. Indirect pathways, although hypothesized, have not been well established^4^. We do not know the effect of heat on maternal stress or on health system functioning, both of which are important in sub-Saharan Africa. The effect of ambient temperature on healthcare workers’ performance in sub-Saharan Africa has received little attention but could also contribute to stillbirths. A recent study from Australia points to increased fatigue in surgeons exposed to temperature of 31 °C even with limited exposure of 2–3 h (ref. ^48^). Heat exposure of healthcare workers may, thus, exacerbate further quality-of-care deficiencies in these settings where labor rooms are not temperature controlled.

Our findings highlight the need to consider improved protection of pregnant and laboring women from adverse effects of rising temperatures. Early warning systems and health promotion recommendations are warranted, such as use of shaded environments as outlined by experts^4,11^. We would like to highlight that appropriate and timely obstetric management has the potential to nearly eliminate intrapartum stillbirths, which is why this study underscores the importance of quality intrapartum care. In response, the concept of respectful, responsive and high-quality intrapartum care is even more relevant to mitigate heat-related deaths. Additional measures may include re-designing clinics and labor wards to reduce heat impacts through improved construction, allowing air flow and roof insulation, combined with the creation of green spaces near the labor ward^49^.

Our results provide support for an increased risk of perinatal mortality with heat exposure during the last week of pregnancy in sub-Saharan Africa. Although our findings are not conclusive for all estimates, we provide much-needed evidence from a region where, to date, few empirical studies are published but where climate change and increasing temperatures are of particular concern. Increased efforts are needed to improve the quality of intrapartum care to ensure that hard-won improvements in mortality in sub-Saharan Africa are not reversing due to heat exposure. Furthermore, implementation research is needed to develop appropriate interventions to protect pregnant women from the negative effects of extreme heat.

### Ethics and inclusion statement

The ALERT study was approved by local and national institutional review boards with details provided in our protocol paper^50^. It was approved by the following: Karolinska Institutet, Sweden (Etikprövningsmyndigheten—Dnr 2020–01587); Uganda National Council for Science and Technology (UNCST)—(HS1324ES); Muhimbili University of Health and Allied Sciences (MUHAS) Research and Ethics Committee, Tanzania (MUHAS-REC-04-2020-118); Aga Khan University Ethical Review Committee, Tanzania (AKU/2019/044/fb); College of Medicine Research and Ethics Committee (COMREC), Malawi—(COMREC P.04/20/3038); Comité National d’Ethique pour la Recherche en Santé, Cotonou, Bénin—(83/MS/DC/SGM/CNERS/ST); the institutional review board at the Institute of Tropical Medicine Antwerp; and the Ethics Committee at the University Hospital Antwerp, Belgium—(ITG 1375/20. B3002020000116).

Approval from all participating countries exempted the study from requiring individual informed consent from women and their fetus, as only routinely documented and abstracted data were collected in the ALERT perinatal e-registry. This is a common approach also done in high-income countries, such as Sweden^51^, to address difficulties to obtain consent during labor^52^. Data were de-identified and did not include any socio-demographic information of wealth, education or place of living, which would have required consent. This guaranteed meticulous protection of privacy and confidentiality for all participants.

The ALERT consortium is committed to equitable inclusion and authorship. The authorship list includes all ALERT members who conceptualized the trial and supported the data collection, including quality assurance and processing of the data. Authorship and contributions are discussed during monthly consortium meetings, which include all members. All publications are internally reviewed before submission as outlined in our ALERT publication and data use policy. Authorship of key analysis papers involving all four countries are discussed within the ALERT steering committee meetings involving the country principal investigators and work package leaders. Our ALERT consortium includes three postdoctoral researchers, six PhD students and over 15 MSc students, supporting our strong capacity-building commitment. Most MSc students, PhD students and postdoctoral fellows are from low-resource settings and are female. Local knowledge is taken into consideration throughout the study implementation through co-design and work package meetings. An increasing number of papers are being published on issues relevant to ALERT members—see https://alert.ki.se/.

The idea of this study was born out of the increased recognition and interest of the effect of climate change and increasing temperature within the ALERT consortium. Conceptualization and methodological approaches of the present study were discussed during Zoom meetings including all authors and has sparked further discussions and development of academic work.

## Methods

### Data source and study population

This study included singleton births from a prospective observational study in 16 hospitals in Benin, Malawi, Tanzania and Uganda, which were collected as part of the Action Leveraging Evidence to Reduce perinatal morTality and morbidity (ALERT) study^50^. Benin, Malawi, Tanzania and Uganda are low-income and lower-middle-income countries facing a large perinatal mortality burden^1^. They are categorized as low-income or lower-middle-income countries with real gross domestic product per capita of between US$1,500 (Malawi) and US$3,300 (Benin) (Supplementary Table 1). Uptake to antenatal and childbirth care in facilities is improving, but health systems are weak. Childbirth care faces larger out-of-pocket expenses, particularly in Benin and Uganda, restricting preventive uptake of hospital care.

In each of the countries, four medium-size hospitals were included with more than 2,500 births per annum. Hospitals were typically district or regional public and private-non-profit (faith-based) facilities, although the national referral hospital in Cotonou, Benin, also took part in the study. In this analysis, we included all mother–baby pairs admitted for childbirth in any of the hospitals between 1 July 2021 and 31 December 2023. We excluded mother–baby pairs who were referred to the hospitals after giving birth. Data were entered daily by data clerks who were nurses or midwives in the maternity ward of each hospital. An initial 2-day training, subsequent regular on-site supervision and a WhatsApp group supported the data collection. Data were abstracted from standardized paper-based case notes and abstracted into a pre-programmed harmonized perinatal e-registry using the Research Electronic Data Capture (REDCap) platform. The application had in-build checks for completeness, ranges and consistencies. Initially weekly, later monthly, Zoom calls checked for completeness of the data entry. Furthermore, prepared do-files preparing initial analysis allowed the follow-up of a larger number of indicators to critically review face validity; protocol details are published elsewhere^53^. Observations with missing outcome (*n* = 85) and exposure (*n* = 145) data were removed from the dataset (Supplementary Fig. 1).

ALERT is registered (17 June 2020) in the Pan African Clinical Trial Registry at 202006793783148. The ALERT study was approved by the Swedish Ethical Authority and in each participating country, with details provided in our protocol paper^50^. Approval from all participating countries exempted the study from informed individual consent from women and their fetuses, as only routinely documented and abstracted data were used in the ALERT perinatal e-registry, and data were de-identified.

### Outcome assessment

Our main outcomes were stillbirths (including both antepartum and intrapartum deaths) and very early neonatal deaths documented in REDCap by nurses or midwives in each hospital^53^. Stillbirths were defined as the death of a fetus before birth, weighing at least 1,000 g, and which could not be resuscitated after birth. Perinatal deaths included both stillbirths and deaths within the first 24 h (very early neonatal deaths). We chose 24 h as a cutoff as mothers were usually discharged the day after birth. Data collectors and nursing staff in the study hospitals were trained to distinguish antepartum and intrapartum stillbirth based on skin appearance rather than in relation to onset of labor. Because skin disintegration is considered to begin 12 h after death, macerated stillbirth is assumed to have occurred before the onset of labor, and fresh stillbirth after, and these were used as a proxy for antepartum and intrapartum stillbirth, respectively. Training protocols followed national standards. Our investigation into the data confirmed that data are more reliable when using the physical appearance. First, fetal heart rate is more often not reported for macerated stillbirths; health providers may be unsure if the fetal heart rate is not present and then decide not to document at all. Second, women often reach the hospital late in labor, particularly if they are referred, which is why a negative fetal heart beat at admission cannot be used as a clear indication that the baby was already dead when labor started.

### Other obstetric variables for stratified analysis

For the stratified analysis, we considered obstetric variables that were collected as part of our perinatal e-registry. Information was abstracted by nurses or midwives from antenatal care cards (for example, maternal age and HIV status), retrieved from admission files (for example, complications such as hypertensive disorders and antepartum bleeding) or clinical files (for example, babies’ sex and birth weight) and entered into the electronic REDCap perinatal e-registry form^53^.

### Exposure assessment

We obtained daily mean temperatures at 2 m from the European Centre for Medium-Range Weather Forecasts (ECMWF) at a 9 × 9-km resolution (0.1° × 0.1°). Data were freely downloaded from the Copernicus Climate Data store (https://cds.climate.copernicus.eu/). We linked the daily mean temperatures at the specific grid of ECMWF within which each hospital was located and estimated the daily mean temperature for each day during the last week of each pregnancy (lag 0–6 d). We further extracted 24-hourly data from the ECMWG to estimate the mean, maximum and minimum values. In addition, we included daily dew point temperature at 2 m obtained from the ECMWF to estimate relative humidity using the Magnus formula, which incorporates both air and dew point temperatures^54^.

### Statistical analysis

We estimated the association between multiple short-term air temperature metrics the week before birth (lag 0–6 d) and perinatal mortality using a case–crossover design. This design has been widely used in short-term studies, including for the association between temperature and perinatal mortality^55^. The major advantage of the case–crossover design is that each case serves as its own control, allowing for precise adjustment of individual-level confounders that remain constant or change slowly over time, such as age and obstetric risk factors^55^. In our study, we adopted a bi-directional asymmetrical time-stratified strategy to select control days for each case that has been shown to provide unbiased estimates^45^. This strategy involves defining control days as the same day of the week within the same month as childbirth. By employing this approach, we can effectively control for day of the week and other short-term temporal trends, as the cases and controls are closely matched in time. Additionally, the separation of cases and controls by 7 d helps reduce serial autocorrelation in exposures and outcomes^55^. We first applied country-specific distributed-lag nonlinear models with the conditional logistic regression to evaluate the association between temperature metrics the week before birth (lag 0–6 d) and perinatal mortality. Previous studies found consistent nonlinear relationships (U-shaped and J-shaped) between temperature and mortality outcomes^56,57^. To account for this nonlinearity, we applied a natural cubic spline with three internal knots placed at the 10th, 75th and 90th percentiles of country-specific temperature distributions and the lag-response curve with a natural cubic spline with three internal knots placed at equally spaced values in the log scale. This selection was based on previous literature to capture the effect of warm temperatures^37,58^. We then summarized the risk for heat, computing the ORs and 95% CIs and comparing the 99th percentile versus the 75th percentile of the annual temperature distribution. We applied this percentile to accurately reflect the warm season, as the estimated minimum mortality temperature (MMT) in some countries was set at very low levels of the temperature distribution. The MMT was evaluated as the lowest point in the temperature range from the country-specific temperature–perinatal mortality curves^59^. We considered the week before the birth period (lag 0–6 d) as our reference exposures because previous studies indicated this to be the period of highest susceptibility^23,28,37,38^. In the second stage, we applied a random-effects meta-analytical model to pool the country-specific estimates of associations of temperature and perinatal deaths. We applied meta-analyses rather than pooled analysis to account for country-specific differences, such as demographic characteristics, socioeconomic status, healthcare infrastructure, environmental conditions, cultural practices and levels of perinatal mortality, all of which can influence the magnitude and direction of the observed associations. We calculated *I*^2^ statistics and used Cochran’s Q test to evaluate the between-country heterogeneity. To obtain the overall shape of the association between temperature and perinatal mortality, we applied a multivariate meta-regression to pool the country-specific exposure response using a validated approach used in previous studies^58,60^. Statistical analyses were performed using R software (version 3.6.2).

To evaluate the robustness of our results, we applied several sensitivity analyses. First, we evaluated the effect of temperature 1 week before birth (lag 0–6 d) on stillbirths during the six consecutive hottest months for each country to capture extreme heat events. Second, we explored different lag patterns, including single lag of same day (lag0), 2-d lag (lag 0–1) and 3-d lag (lag 0–2), to identify different windows of vulnerability. Third, to validate our initial selection of knot placements at the 10th, 75th and 90th percentiles, we conducted an evaluation of the heat effect by applying a varying number of knots and adjusting their locations (one knot at the 50th and 75th, two knots at the 25th and 50th, two knots at the 50th and 75th, two knots at the 50th and 90th, two knots at the 75th and 90th, three knots at the 10th, 50th and 90th and three knots at the 25th, 50th and 75th). Fourth, we evaluated the associations of maximum and minimum temperature the week before birth (lag 0–6 d) on perinatal mortality. Fifth, we excluded women who were referred, as the patients may have traveled much greater distances. This non-referred subpopulation may also be closer to the general birthing population. Sixth, we used the 50th percentile as our reference value (in comparison to the 75th value) to evaluate a consistent increase in temperature across countries. Seventh, we adjusted for relative humidity the week before birth (lag 0–6 d). Eighth, we compared our 9 × 9-km resolution temperature values with the coarser 28 × 28-km resolution model, also from the ECMWF, to account for a larger coverage area, as we did not have information about the proximity where the mothers were living from the hospital. Finally, because acute exposures may potentially also be correlated with longer-term exposures, we further evaluated the association between trimester-specific mean temperature exposure and the development of perinatal deaths by applying a time-to-event approach considering gestational age as the time axis. We applied a proportional Cox model adjusted for month of birth, year of conception, sex of the child, maternal age and number of pregnancies. We did not consider this as our main analysis, as our initial hypothesis and analysis plan focused on short-term exposures.

We further stratified the analyses by some key effect modifiers that could potentially modify the associations between short-term exposure of ambient air pollution and still births. The key modifiers included maternal age, HIV status, hypertensive disorders, number of pregnancies, sex of the child, preterm births, low birth weight, labor complication and antepartum hemorrhage.

### Reporting summary

Further information on research design is available in the Nature Portfolio Reporting Summary linked to this article.

## Online content

Any methods, additional references, Nature Portfolio reporting summaries, source data, extended data, supplementary information, acknowledgements, peer review information; details of author contributions and competing interests; and statements of data and code availability are available at 10.1038/s41591-024-03245-7.

## Supplementary information

Supplementary Information Supplementary Tables 1–3 and Figs. 1 and 2.

Reporting Summary

Supplementary Data 1 Analysis codes—descriptive analysis.

Supplementary Data 2 Analysis codes—results.

## Data Availability

The perinatal e-registry data will be made publicly available 3 years after the finalization of the trial—thus, as of 31 December 2027—as outlined in our data management plan and publication policy. The data are available earlier upon reasonable request, and any person wanting to use the data before this date will need to request to the ALERT steering committee headed by the corresponding author. Access will be restricted if ALERT members are carrying out or planning a similar analysis. The ALERT steering committee will handle the request and provide a response within 1 month. If the ALERT steering committee gives a positive decision, data will be released promptly. The temperature data are freely available from the Copernicus Climate Data store (https://cds.climate.copernicus.eu/).

## Code availability

The codes are uploaded on GitHub: https://github.com/jdebont/Heat_stillbirth_Hanson_deBont_NatureMedicine_Rcodes.

## References

[1] UNICEF. Levels and trends in child mortality. https://data.unicef.org/resources/levels-and-trends-in-child-mortality-2024/ (2024).

[2] United Nations. The 17 Goals. https://sdgs.un.org/goals (2024).

[3] World Health Organization. Every Newborn Action Plan. https://www.who.int/initiatives/every-newborn-action-plan#:~:text=The%20global%20Every%20Newborn%20Action,reducing%20maternal%20mortality%20and%20morbidity (2014).

[4] Roos, N. et al. Maternal and newborn health risks of climate change: a call for awareness and global action. *Acta Obstet. Gynecol. Scand.* **100**, 566–570 (2021). 33570773 10.1111/aogs.14124

[5] IPCC Climate Change 2022: Impacts, Adaptation and Vulnerability (eds Pörtner, H.-O. et al.) (Cambridge Univ. Press, 2022).

[6] World Meteorological Organization. Africa suffers disproportionately from climate change. https://wmo.int/media/news/africa-suffers-disproportionately-from-climate-change (4 September 2023).

[7] Chersich, M. F. et al. Associations between high temperatures in pregnancy and risk of preterm birth, low birth weight, and stillbirths: systematic review and meta-analysis. *BMJ* **371**, m3811 (2020). 33148618 10.1136/bmj.m3811 PMC7610201

[8] Syed, S., O’Sullivan, T. L. & Phillips, K. P. Extreme heat and pregnancy outcomes: a scoping review of the epidemiological evidence. *Int. J. Environ. Res. Public Health* **19**, 2412 (2022). 35206601 10.3390/ijerph19042412 PMC8874707

[9] Dalugoda, Y., Kuppa, J., Phung, H., Rutherford, S. & Phung, D. Effect of elevated ambient temperature on maternal, foetal, and neonatal outcomes: a scoping review. *Int. J. Environ. Res. Public Health* **19**, 1771 (2022). 10.3390/ijerph19031771 PMC8835067 35162797

[10] Samuels, L. et al. Physiological mechanisms of the impact of heat during pregnancy and the clinical implications: review of the evidence from an expert group meeting. *Int. J. Biometeorol.* **66**, 1505–1513 (2022). 35554684 10.1007/s00484-022-02301-6 PMC9300488

[11] Bonell, A. et al. An expert review of environmental heat exposure and stillbirth in the face of climate change: clinical implications and priority issues. *BJOG* **131**, 623–631 (2023). 10.1111/1471-0528.17622 37501633

[12] Rekha, S. et al. Heat stress and adverse pregnancy outcome: prospective cohort study. *BJOG* **131**, 612–622 (2024). 37814395 10.1111/1471-0528.17680

[13] Shashar, S. et al. Temperature and preeclampsia: epidemiological evidence that perturbation in maternal heat homeostasis affects pregnancy outcome. *PLoS ONE* **15**, e0232877 (2020). 32421729 10.1371/journal.pone.0232877 PMC7234374

[14] Part, C. et al. Ambient temperature during pregnancy and risk of maternal hypertensive disorders: a time-to-event study in Johannesburg, South Africa. *Environ. Res.* **212**, 113596 (2022). 35661733 10.1016/j.envres.2022.113596

[15] He, S., Kosatsky, T., Smargiassi, A., Bilodeau-Bertrand, M. & Auger, N. Heat and pregnancy-related emergencies: risk of placental abruption during hot weather. *Environ. Int.* **111**, 295–300 (2018). 29146008 10.1016/j.envint.2017.11.004

[16] Rammah, A. et al. Temperature, placental abruption and stillbirth. *Environ. Int.* **131**, 105067 (2019). 31376592 10.1016/j.envint.2019.105067 PMC11024926

[17] Bonell, A. et al. A protocol for an observational cohort study of heat strain and its effect on fetal wellbeing in pregnant farmers in The Gambia. *Wellcome Open Res.* **5**, 32 (2020). 32292825 10.12688/wellcomeopenres.15731.1 PMC7141168

[18] Haghighi, M. M. et al. Impacts of high environmental temperatures on congenital anomalies: a systematic review. *Int. J. Environ. Res. Public Health* **18**, 4910 (2021). 10.3390/ijerph18094910 PMC8124753 34063033

[19] Aminu, M. et al. Causes of and factors associated with stillbirth in low- and middle-income countries: a systematic literature review. *BJOG* **121**, 141–153 (2014). 25236649 10.1111/1471-0528.12995

[20] Wells, J. C. Thermal environment and human birth weight. *J. Theor. Biol.* **214**, 413–425 (2002). 11846599 10.1006/jtbi.2001.2465

[21] de Bont, J. et al. Associations between ambient temperature and risk of preterm birth in Sweden: a comparison of analytical approaches. *Environ. Res.* **213**, 113586 (2022). 35671796 10.1016/j.envres.2022.113586

[22] Nakstad, B. et al. How climate change may threaten progress in neonatal health in the African region. *Neonatology* **119**, 644–651 (2022). 10.1159/000525573 35850106

[23] Basu, R., Sarovar, V. & Malig, B. J. Association between high ambient temperature and risk of stillbirth in California. *Am. J. Epidemiol.* **183**, 894–901 (2016). 27037268 10.1093/aje/kwv295

[24] Kanner, J. et al. Ambient temperature and stillbirth: risks associated with chronic extreme temperature and acute temperature change. *Environ. Res.* **189**, 109958 (2020). 32980027 10.1016/j.envres.2020.109958

[25] UNICEF. Never forgotten. The situation of stillbirth around the globe. https://data.unicef.org/resources/never-forgotten-stillbirth-estimates-report/ (2023).

[26] Flenady, V. et al. Major risk factors for stillbirth in high-income countries: a systematic review and meta-analysis. *Lancet* **377**, 1331–1340 (2011). 21496916 10.1016/S0140-6736(10)62233-7

[27] Auger, N., Fraser, W. D., Smargiassi, A., Bilodeau-Bertrand, M. & Kosatsky, T. Elevated outdoor temperatures and risk of stillbirth. *Int. J. Epidemiol.* **46**, 200–208 (2017). 27160765 10.1093/ije/dyw077 PMC5837696

[28] Ha, S. et al. Ambient temperature and stillbirth: a multi-center retrospective cohort study. *Environ. Health Perspect.* **125**, 067011 (2017). 28650842 10.1289/EHP945 PMC5743476

[29] Sexton, J. et al. Systematic review of ambient temperature exposure during pregnancy and stillbirth: methods and evidence. *Environ. Res.* **197**, 111037 (2021). 33781772 10.1016/j.envres.2021.111037

[30] Gage, A. D., Fink, G., Ataguba, J. E. & Kruk, M. E. Hospital delivery and neonatal mortality in 37 countries in sub-Saharan Africa and South Asia: an ecological study. *PLoS Med.* **18**, e1003843 (2021). 34851947 10.1371/journal.pmed.1003843 PMC8635398

[31] McElroy, S., Ilango, S., Dimitrova, A., Gershunov, A. & Benmarhnia, T. Extreme heat, preterm birth, and stillbirth: a global analysis across 14 lower-middle income countries. *Environ. Int.* **158**, 106902 (2022). 34627013 10.1016/j.envint.2021.106902

[32] Ranjbaran, M. et al. Effect of ambient air pollution and temperature on the risk of stillbirth: a distributed lag nonlinear time series analysis. *J. Environ. Health Sci. Eng.* **18**, 1289–1299 (2020). 33312643 10.1007/s40201-020-00547-z PMC7721760

[33] Khodadadi, N., Dastoorpoor, M., Khanjani, N. & Ghasemi, A. Universal Thermal Climate Index (UTCI) and adverse pregnancy outcomes in Ahvaz, Iran. *Reprod. Health* **19**, 33 (2022). 35109854 10.1186/s12978-022-01344-7 PMC8811963

[34] Savitz, D. A. & Hu, H. Ambient heat and stillbirth in Northern and Central Florida. *Environ. Res.* **199**, 111262 (2021). 33974845 10.1016/j.envres.2021.111262 PMC8638076

[35] Nyadanu, S. D. et al. Prenatal exposure to long-term heat stress and stillbirth in Ghana: a within-space time-series analysis. *Environ. Res.* **222**, 115385 (2023). 36736550 10.1016/j.envres.2023.115385

[36] Yang, H. Y., Lee, J. K. W. & Chio, C. P. Extreme temperature increases the risk of stillbirth in the third trimester of pregnancy. *Sci. Rep.* **12**, 18474 (2022). 36323816 10.1038/s41598-022-23155-3 PMC9630541

[37] Li, S., Chen, G., Jaakkola, J. J. K., Williams, G. & Guo, Y. Temporal change in the impacts of ambient temperature on preterm birth and stillbirth: Brisbane, 1994–2013. *Sci. Total Environ.* **634**, 579–585 (2018). 29635200 10.1016/j.scitotenv.2018.03.385

[38] Bekkar, B., Pacheco, S., Basu, R. & DeNicola, N. Association of air pollution and heat exposure with preterm birth, low birth weight, and stillbirth in the US: a systematic review. *JAMA Netw. Open* **3**, e208243 (2020). 32556259 10.1001/jamanetworkopen.2020.8243 PMC7303808

[39] Asamoah, B., Kjellstrom, T. & Östergren, P. O. Is ambient heat exposure levels associated with miscarriage or stillbirths in hot regions? A cross-sectional study using survey data from the Ghana Maternal Health Survey 2007. *Int. J. Biometeorol.* **62**, 319–330 (2018). 28748383 10.1007/s00484-017-1402-5 PMC5854714

[40] Caniglia, E. C. et al. Seasonality of adverse birth outcomes in women with and without HIV in a representative birth outcomes surveillance study in Botswana. *BMJ Open* **11**, e045882 (2021). 34479931 10.1136/bmjopen-2020-045882 PMC8420660

[41] Amouzou, A. et al. Health service utilisation during the COVID-19 pandemic in sub-Saharan Africa in 2020: a multicountry empirical assessment with a focus on maternal, newborn and child health services. *BMJ Glob. Health* **7**, e008069 (2022). 35501068 10.1136/bmjgh-2021-008069 PMC9062456

[42] Semaan, A. et al. Provision and utilization of maternal health services during the COVID-19 pandemic in 16 hospitals in sub-Saharan Africa. *Front. Glob. Womens Health* **4**, 1192473 (2023). 38025986 10.3389/fgwh.2023.1192473 PMC10644718

[43] Hanson, C. et al. Stillbirth mortality by Robson ten-group classification system: a cross-sectional registry of 80 663 births from 16 hospital in sub-Saharan Africa. *BJOG*10.1111/1471-0528.17833 (2024). 10.1111/1471-0528.17833 38725396

[44] Straneo, M. et al. Inequalities in use of hospitals for childbirth among rural women in sub-Saharan Africa: a comparative analysis of 18 countries using demographic and health survey data. *BMJ Glob. Health* **9**, e013029 (2024). 38262683 10.1136/bmjgh-2023-013029 PMC10806834

[45] Janes, H., Sheppard, L. & Lumley, T. Case–crossover analyses of air pollution exposure data: referent selection strategies and their implications for bias. *Epidemiology* **16**, 717–726 (2005). 16222160 10.1097/01.ede.0000181315.18836.9d

[46] Hanson, C. et al. Maternal mortality and distance to facility-based obstetric care in rural southern Tanzania: a secondary analysis of cross-sectional census data in 226 000 households. *Lancet Glob. Health* **3**, e387–e395 (2015). 26004775 10.1016/S2214-109X(15)00048-0

[47] Coughlan de Perez, E., Arrighi, J. & Marunye, J. Challenging the universality of heatwave definitions: gridded temperature discrepancies across climate regions. *Clim. Change* **176**, 167 (2023).

[48] Palejwala, Z. et al. Higher operating theatre temperature during burn surgery increases physiological heat strain, subjective workload, and fatigue of surgical staff. *PLoS ONE* **18**, e0286746 (2023). 37267345 10.1371/journal.pone.0286746 PMC10237492

[49] Kuehn, L. & McCormick, S. Heat exposure and maternal health in the face of climate change. *Int. J. Environ. Res. Public Health* **14**, 853 (2017). 28758917 10.3390/ijerph14080853 PMC5580557

[50] Akuze, J. et al. Action leveraging evidence to reduce perinatal mortality and morbidity (ALERT): study protocol for a stepped-wedge cluster-randomised trial in Benin, Malawi, Tanzania and Uganda. *BMC Health Serv. Res.* **21**, 1324 (2021). 34895216 10.1186/s12913-021-07155-z PMC8665312

[51] Stephansson, O., Petersson, K., Björk, C., Conner, P. & Wikström, A.-K. The Swedish Pregnancy Register—for quality of care improvement and research. *Acta Obstet. Gynecol. Scand.* **97**, 466–476 (2018). 29172245 10.1111/aogs.13266 PMC5873375

[52] Widmer, M., Bonet, M. & Betrán, A. P. Would you like to participate in this trial? The practice of informed consent in intrapartum research in the last 30 years. *PLoS ONE* **15**, e0228063 (2020). 31978100 10.1371/journal.pone.0228063 PMC6980544

[53] Abeid, M. et al. Report of a prototype for a perinatal e-registry. https://cordis.europa.eu/project/id/847824/results (European Commission, 2021).

[54] Wu, Y. et al. Effects of temperature and humidity on the daily new cases and new deaths of COVID-19 in 166 countries. *Sci. Total Environ.* **729**, 139051 (2020). 32361460 10.1016/j.scitotenv.2020.139051 PMC7187824

[55] Maclure, M. & Mittleman, M. A. Should we use a case–crossover design? *Annu. Rev. Public Health* **21**, 193–221 (2000). 10884952 10.1146/annurev.publhealth.21.1.193

[56] Gasparrini, A. et al. Mortality risk attributable to high and low ambient temperature: a multicountry observational study. *Lancet* **386**, 369–375 (2015). 26003380 10.1016/S0140-6736(14)62114-0 PMC4521077

[57] Chen, R. et al. Association between ambient temperature and mortality risk and burden: time series study in 272 main Chinese cities. *BMJ* **363**, k4306 (2018). 30381293 10.1136/bmj.k4306 PMC6207921

[58] Gasparrini, A. & Armstrong, B. Reducing and meta-analysing estimates from distributed lag non-linear models. *BMC Med. Res. Methodol.* **13**, 1 (2013). 23297754 10.1186/1471-2288-13-1 PMC3599933

[59] Liu, J. et al. Mortality burden attributable to high and low ambient temperatures in China and its provinces: results from the Global Burden of Disease Study 2019. *Lancet Reg. Health West. Pac.* **24**, 100493(2022). 10.1016/j.lanwpc.2022.100493 PMC9213765 35756888

[60] Gasparrini, A., Armstrong, B. & Kenward, M. G. Multivariate meta-analysis for non-linear and other multi-parameter associations. *Stat. Med.* **31**, 3821–3839 (2012). 22807043 10.1002/sim.5471 PMC3546395

