# Supplementary material for: A time-stratified, case–crossover study of heat exposure and perinatal mortality from 16 hospitals in sub-Saharan Africa
Source: Nat Med. 2024 Sep 3;30(11):3106–13. doi: 10.1038/s41591-024-03245-7 (PMC11564089; doi:10.1038/s41591-024-03245-7)
Supplement: Supplementary file 1 — Supplementary Tables 1–3 and Figs. 1 and 2. [file 41591_2024_3245_MOESM1_ESM.pdf]

nature medicine

Supplementary information https://doi.org/10.1038/s41591-024-03245-7

A time-stratified, case–crossover study of heat exposure and perinatal mortality from 16 hospitals in sub-Saharan Africa

In the format provided by the authors and unedited

Supplementary material

Supplement Table S1. Socio-economic and health system of the included countries
Supplement Table S2. Associations between temperature indicators the week before birth (lag 0-6) and perinatal mortality (75th vs 99th percentile).
Supplement Table S3. Associations between temperature indicators the week before birth (lag 0-6) and perinatal mortality (50th vs 99th percentile).
Supplement Fig. S1. Flowchart of the study population.
Supplement Fig. S2. Distribution of the temperature indicators (average levels the week before birth) by country.

Table S1. Socio-economic and health system of the included countries

**Indicator**	Benin	Malawi	Tanzania	Uganda
**National Indicators**				
Region in Africa	West	East	East	East
Real Gross Domestic Product Per Capita (United States Dollar) (2021) [1]	3,300	1,500	2,600	2,200
Population (million) (2023) [1]	14.2	21.3	65.6	47.7
**Neonatal Health Indicators**				
Neonatal Mortality Rate (per 1,000 live births) (2021) [2]	29	19	20	19
Stillbirth Rate (per 1,000 live births) (2021) [2]	20	16	18	15
**Maternal Health Indicators**				
Maternal Mortality Ratio (per 100,000 live births) (2020) [3]	523	381	238	284
Antenatal Care[4]				
≥1 Visits (%)	83%	98%	90%	95%
≥4 Visits (%)	52%	51%	65%	57%
Deliveries by Skilled Birth Attendant (%)[4]	78%	90%	85%	74%
Institutional Deliveries (%)[4]	84%	91%	81%	73%
Cesarean Section Deliveries (%)[4]	5%	6%	11%	6%
Official User Fees for Childbirth (2021)[4]	Yes	No	No	No
Total Fertility Rate	4.8	3.8	4.8	4.4
Unmet Need for Family Planning[5]	26%	15%	21%	20%
**Health System Indicators**[6]				
Medical Doctors (per 10,000 population) (2019)	0.6	0.5	0.5	1.6
Nursing and Midwifery (per 10,000 population) (2018)	2.9	7.0	16.9	5.6
Current Health Expenditure per Capita (USD) (2021)	35.13	46.56	37.16	43.45
Current Health Expenditure as % of Gross Domestic Product (2021)	2.6%	7.4%	3.4%	4.7%
Out of Pocket Expenditure as a % of Current Health Expenditure (2021)	48.6%	14.1%	25.6%	31.3%

Sources:
1.CIA. The Global Factbook. 2024. https://www.cia.gov/the-world-factbook/africa/ (accessed 10 Mar 2024).
2.UN Inter-agency Group for Child Mortality Estimation. Levels and Trends in Child Mortality. 2024. https://childmortality.org/ (accessed 10 March 2024).
3.World Health Organization. Trends in maternal mortality 2000 to 2020: estimates by WHO, UNICEF, UNFPA, World Bank Group and UNDESA/Population Division. Geneva, 2023.
4.UNICEF. UNICEF Data: Monitoring the situation of children and women 2023. https://data.unicef.org/ (accessed 24 March 2024) & DHS data 2022 for Tanzania https://dhsprogram.com/publications/publication-FR382-DHS-Final-Reports.cfm (assessed 24 March 2024)
5.UNFPA. Population Data Portal. 2023. https://pdp.unfpa.org/?_ga=2.127365005.1866037946.1710834186-817427990.1710834186&country=204&data_id=dataSource_8-6%3A23%2CdataSource_8-1%3A2%2B10%2B11%2CdataSource_8-2%3A721%2CdataSource_8-5%3A19238&page=Explore-Indicators
(accessed 24 March 2024).
6.World Health Organization. Global Health Observatory (GHO) data. https://www.who.int/data/gho/data/indicators (accessed 10 March 2024).

Supplement Table S2| Associations between temperature indicators the week before birth (lag 0-6) and perinatal mortality (75th vs 99th percentile).

	Stillbirth		Antepartum stillbirth		Intrapartum stillbirth		Perinatal death	
**Country**	**75th (°C)**	**OR (95%CI)**	**75th (°C)**	**OR (95%CI)**	**75th (°C)**	**OR (95%CI)**	**75th (°C)**	**OR (95%CI)**
**Mean temperature**								
Benin	28.0	1.14 (0.65; 2.01)	27.9	1.53 (0.60; 3.88)	28.0	0.71 (0.34; 1.48)	28.0	1.30 (0.76; 2.20)
Malawi	22.1	1.66 (0.83; 3.33)	22.2	1.10 (0.40; 3.03)	22.0	2.81 (1.06; 7.41)	22.5	1.39 (0.76; 2.52)
Tanzania	25.9	0.94 (0.30; 2.92)	25.9	0.40 (0.07; 2.15)	25.8	9.03 (1.01; 80.42)	26.0	1.03 (0.39; 2.69)
Uganda	22.7	1.32 (0.82; 2.15)	22.8	1.31 (0.58; 2.97)	22.7	1.39 (0.74; 2.61)	22.7	1.42 (0.91; 2.22)
**Meta-analysis**		**1.29 (0.95; 1.77)**		**1.18 (0.71; 1.95)**		**1.64 (0.74; 3.63)**		**1.34 (1.01; 1.78)**
**Max temperature**								
Benin	31.4	1.07 (0.67; 1.71)	31.3	1.01 (0.52; 1.99)	31.4	1.05 (0.55; 2.00)	31.1	1.10 (0.71; 1.70)
Malawi	27.7	2.10 (0.97; 4.55)	27.8	1.87 (0.60; 5.84)	27.6	3.02 (0.98; 9.25)	28.2	1.83 (0.94; 3.59)
Tanzania	31.9	0.57 (0.14; 2.32)	31.9	0.18 (0.02; 1.66)	31.7	3.72 (0.41; 33.88)	31.7	0.56 (0.16; 1.93)
Uganda	26.8	1.24 (0.70; 2.21)	26.8	1.35 (0.53; 3.47)	26.8	1.01 (0.46; 2.19)	27.0	1.35 (0.79; 2.28)
**Meta-analysis**		**1.22 (0.89; 1.68)**		**1.12 (0.69; 1.82)**		**1.33 (0.81; 2.20)**		**1.24 (0.93; 1.66)**
**Min temperature**								
Benin	25.9	1.36 (0.68; 2.69)	25.8	1.64 (0.55; 4.89)	26.0	1.23 (0.52; 2.87)	25.9	1.57 (0.85; 2.91)
Malawi	17.9	1.27 (0.59; 2.74)	18.0	0.97 (0.32; 2.94)	17.8	1.56 (0.54; 4.47)	18.0	1.07 (0.51; 2.25)
Tanzania	22.0	1.37 (0.37; 5.11)	22.1	0.69 (0.11; 4.34)	22.0	6.24 (0.52; 75.35)	22.1	1.31 (0.38; 4.53)
Uganda	19.4	1.26 (0.62; 2.57)	19.4	0.49 (0.16; 1.57)	19.4	2.33 (0.97; 5.59)	19.3	1.21 (0.62; 2.36)
**Meta-analysis**		**1.30 (0.88; 1.94)**		**0.91 (0.50; 1.68)**		**1.74 (1.04; 2.92)**		**1.30 (0.90; 1.88)**

Note: Country-specific and pooled estimates are presented as odds ratio (OR) and 95% confidence intervals obtained from conditional logistic regression models. The estimates show the association of an increase in mean, maximum and minimum temperature (from the 75th to the 99th percentile) the week before birth (lag 0-6) on stillbirths, antepartum stillbirths, intrapartum stillbirths, and perinatal deaths. The data were obtained from 16 hospitals in four different countries, including Benin (4 hospitals), Malawi (4), Tanzania (4), and Uganda (4). The absolute temperature value (°C) corresponds to the 75th percentile for each country, temperature indicator and outcome.

Supplement Table S3| Associations between temperature indicators the week before birth (lag 0-6) and perinatal mortality (50th vs 99th percentile).

Country	Stillbirth		Antepartum stillbirths		Intrapartum stillbirths		Perinatal death	
	50th (°C)	OR (95%CI)	50th (°C)	OR (95%CI)	50th (°C)	OR (95%CI)	50th (°C)	OR (95%CI)
**Mean temperature**								
Benin	27.1	0.95 (0.53; 1.71)	27.0	1.20 (0.45; 3.16)	27.2	0.63 (0.30; 1.34)	27.1	1.09 (0.63; 1.89)
Malawi	20.1	1.34 (0.65; 2.74)	20.2	1.05 (0.37; 3.00)	20.0	1.81 (0.66; 5.00)	20.1	1.11 (0.60; 2.07)
Tanzania	24.6	1.04 (0.35; 3.09)	24.6	0.38 (0.07; 1.89)	24.7	9.54 (1.17; 77.72)	24.7	1.06 (0.42; 2.67)
Uganda	22.0	1.36 (0.84; 2.22)	22.0	1.31 (0.58; 2.97)	21.9	1.42 (0.75; 2.69)	22.0	1.46 (0.93; 2.30)
**Meta-analysis**		**1.19 (0.87; 1.64)**		**1.07 (0.65; 1.79)**		**1.41 (0.67; 2.96)**		**1.23 (0.92; 1.64)**
**Max temperature**								
Benin	29.9	1.23 (0.71; 2.12)	29.8	1.03 (0.46; 2.31)	30.0	1.29 (0.61; 2.74)	29.9	1.20 (0.72; 2.02)
Malawi	24.9	1.69 (0.77; 3.70)	25.1	1.65 (0.53; 5.19)	24.8	2.04 (0.66; 6.28)	25.0	1.48 (0.75; 2.92)
Tanzania	29.6	0.53 (0.14; 2.04)	29.6	0.17 (0.02; 1.32)	29.6	3.34 (0.40; 28.04)	29.6	0.54 (0.17; 1.75)
Uganda	25.7	1.27 (0.70; 2.29)	25.8	1.44 (0.56; 3.75)	25.7	0.99 (0.45; 2.18)	25.8	1.38 (0.80; 2.37)
**Meta-analysis**		**1.25 (0.88; 1.76)**		**1.12 (0.66; 1.90)**		**1.33 (0.83; 2.15)**		**1.24 (0.91; 1.71**)
**Min temperature**								
Benin	25.0	1.20 (0.62; 2.33)	24.8	1.49 (0.51; 4.36)	25.1	1.08 (0.47; 2.45)	25.0	1.39 (0.76; 2.55)
Malawi	15.9	1.10 (0.52; 2.35)	16.0	0.91 (0.31; 2.68)	15.8	1.26 (0.44; 3.60)	15.8	0.93 (0.45; 1.89)
Tanzania	20.9	1.34 (0.36; 5.03)	20.9	0.66 (0.11; 4.13)	20.9	4.63 (0.44; 48.71)	21.0	1.21 (0.36; 4.06)
Uganda	18.8	1.30 (0.66; 2.55)	18.8	0.58 (0.19; 1.73)	18.8	2.20 (0.96; 5.04)	18.8	1.25 (0.67; 2.35)
**Meta-analysis**		**1.22 (0.83; 1.78)**		**0.90 (0.50; 1.62)**		**1.54 (0.94; 2.54)**		**1.20 (0.84; 1.71)**

Note: Country-specific and pooled estimates are presented as odds ratio (OR) and 95% confidence intervals obtained from conditional logistic regression models. The estimates show the association of an increase in mean, maximum and minimum temperature (from the 50th to the 99th percentile) the week before birth (lag 0-6) on stillbirths, antepartum stillbirths, intrapartum stillbirths, and perinatal deaths. The data were obtained from 16 hospitals in four different countries, including Benin (4 hospitals), Malawi (4), Tanzania (4), and Uganda (4). The absolute temperature value (°C) corresponds to the 50th percentile for each country, temperature indicator and outcome.

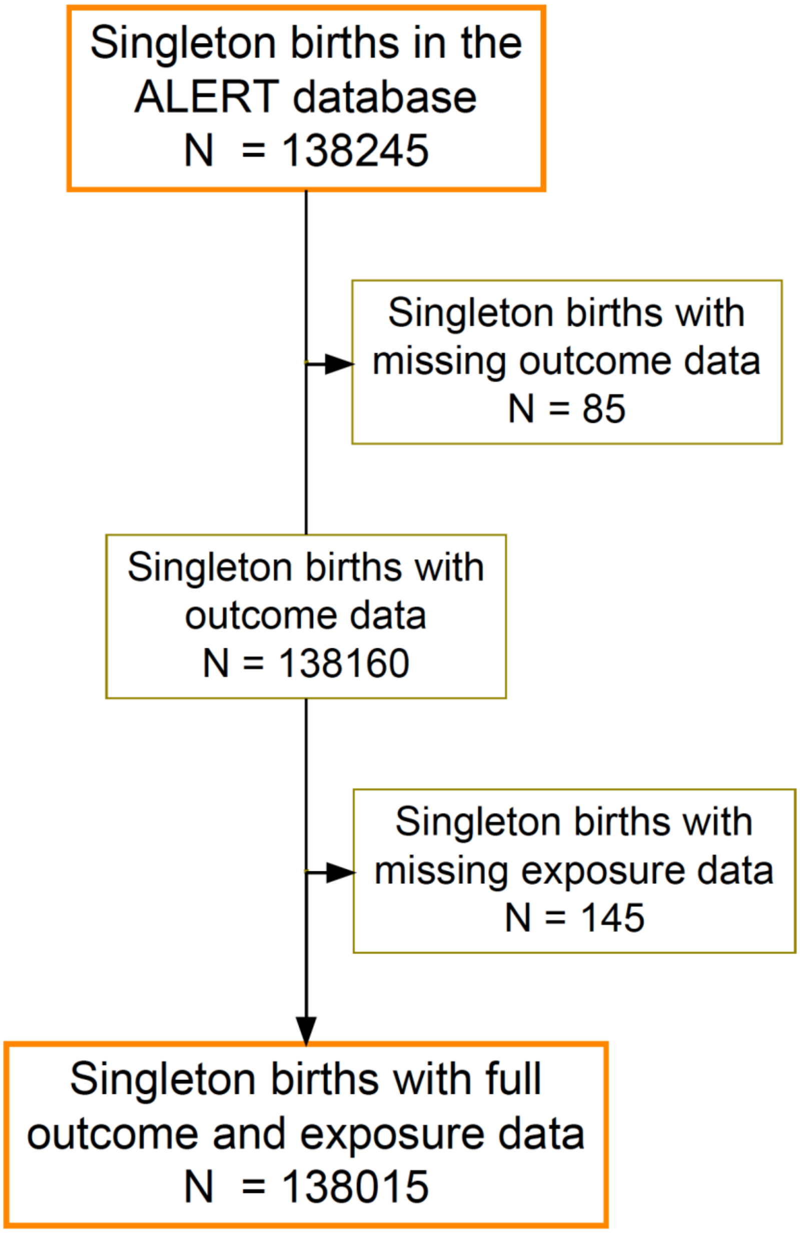

Supplement Fig. S1| Flowchart of the study population.

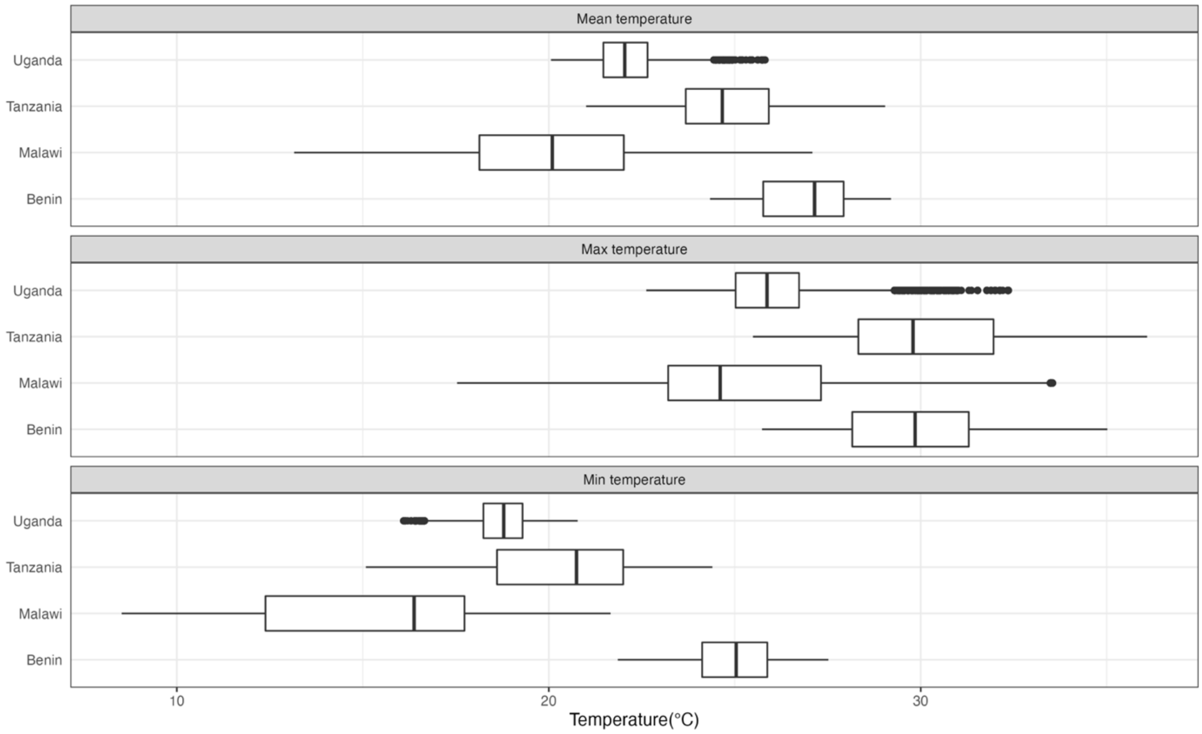

Supplement Fig. S2| Distribution of the temperature indicators (average levels the week before birth) by country.

The figure depicts the mean, maximum and minimum temperatures (°C) average levels the week before birth by country. The boxplot depicting mean, quartiles, minimum, maximum and outliers (>/<1.5x interquartile range).
