# Supplementary material for: A time-stratified, case–crossover study of heat exposure and perinatal mortality from 16 hospitals in sub-Saharan Africa
Source: Nat Med. 2024 Sep 3;30(11):3106–13. doi: 10.1038/s41591-024-03245-7 (PMC11564089; doi:10.1038/s41591-024-03245-7)
Supplement: Supplementary file 2 — Reporting Summary [file 41591_2024_3245_MOESM2_ESM.pdf]

nature portfolio

Corresponding author(s): Claudia Hanson

Last updated by author(s): Jul 12, 2024

Reporting Summary

Nature Portfolio wishes to improve the reproducibility of the work that we publish. This form provides structure for consistency and transparency in reporting. For further information on Nature Portfolio policies, see our Editorial Policies and the Editorial Policy Checklist.

Statistics

For all statistical analyses, confirm that the following items are present in the figure legend, table legend, main text, or Methods section.

n/a	Confirmed	
☐	☒	The exact sample size (*n*) for each experimental group/condition, given as a discrete number and unit of measurement
☐	☒	A statement on whether measurements were taken from distinct samples or whether the same sample was measured repeatedly
☐	☒	The statistical test(s) used AND whether they are one- or two-sided<br>*Only common tests should be described solely by name; describe more complex techniques in the Methods section.*
☐	☒	A description of all covariates tested
☐	☒	A description of any assumptions or corrections, such as tests of normality and adjustment for multiple comparisons
☐	☒	A full description of the statistical parameters including central tendency (e.g. means) or other basic estimates (e.g. regression coefficient) AND variation (e.g. standard deviation) or associated estimates of uncertainty (e.g. confidence intervals)
☐	☒	For null hypothesis testing, the test statistic (e.g. *F*, *t*, *r*) with confidence intervals, effect sizes, degrees of freedom and *P* value noted<br>*Give P values as exact values whenever suitable.*
☒	☐	For Bayesian analysis, information on the choice of priors and Markov chain Monte Carlo settings
☐	☒	For hierarchical and complex designs, identification of the appropriate level for tests and full reporting of outcomes
☐	☒	Estimates of effect sizes (e.g. Cohen's *d*, Pearson's *r*), indicating how they were calculated

Our web collection on statistics for biologists contains articles on many of the points above.

Software and code

Policy information about availability of computer code

Data collection	Research Electronic Data Capture REDCap
Data analysis	R software (version 3.6.2)

For manuscripts utilizing custom algorithms or software that are central to the research but not yet described in published literature, software must be made available to editors and reviewers. We strongly encourage code deposition in a community repository (e.g. GitHub). See the Nature Portfolio guidelines for submitting code & software for further information.

Data

Policy information about availability of data

All manuscripts must include a data availability statement. This statement should provide the following information, where applicable:

- Accession codes, unique identifiers, or web links for publicly available datasets
- A description of any restrictions on data availability
- For clinical datasets or third party data, please ensure that the statement adheres to our policy

The perinatal e-registry data will be made publicly available after the finalization of the trial as of 31 Dec 2027 (three (3) years after finalising the trial) as outlined in our data management plan and publication policy, available on request. Any person wishing to use the data prior to this date will need to place a request to the ALERT steering committee headed by the corresponding author. The temperature data are freely available from the Copernicus Climate Data store (https://cds.climate.copernicus.eu/).

April 2023

Research involving human participants, their data, or biological material

Policy information about studies with human participants or human data. See also policy information about sex, gender (identity/presentation), and sexual orientation and race, ethnicity and racism.

Reporting on sex and gender	Our data are from pregnant women. We further report on the sex of the newborn
Reporting on race, ethnicity, or other socially relevant groupings	no data available. All data are from low and middle income countries in sub-Saharan Africa as reported.
Population characteristics	We detailed the setting (Supplementaty table 1) and the characteristics of the study population (table 1)
Recruitment	We included all mother-baby pairs admitted for childbirth in any of the hospitals between 1st July 2021 and 31st December 2023. We excluded mother-baby pairs who were referred to the hospitals after giving birth.
Ethics oversight	Karolinska Institutet, Sweden (Etikprövningsmyndigheten—Dnr 2020–01587). Uganda National Council for Science and Technology (UNCST)— (HS1324ES). Muhimbili University of Health And Allied Sciences (MUHAS) Research and Ethics Committee, Tanzania (MUHAS-REC-04-2020-118) and The Aga Khan University Ethical Review Committee, Tanzania (AKU/2019/044/fb). College of Medicine Research and Ethics Committee (COMREC), Malawi—(COMREC P.04/20/3038). Comité National d'Ethique pour la Recherche en Santé, Cotonou, Bénin—(83/MS/DC/SGM/CNERS/ST). The Institutional Review Board at the Institute of Tropical Medicine Antwerp and The Ethics Committee at the University Hospital Antwerp, Belgium—(ITG 1375/20. B3002020000116).

Note that full information on the approval of the study protocol must also be provided in the manuscript.

Field-specific reporting

Please select the one below that is the best fit for your research. If you are not sure, read the appropriate sections before making your selection.

☒ Life sciences ☐ Behavioural & social sciences ☐ Ecological, evolutionary & environmental sciences

For a reference copy of the document with all sections, see nature.com/documents/nr-reporting-summary-flat.pdf

Life sciences study design

All studies must disclose on these points even when the disclosure is negative.

Sample size	The sample size was determined by the main trial. See https://bmchealthservres.biomedcentral.com/articles/10.1186/s12913-021-07155-z We used all data available.
Data exclusions	We excluded multiple births. (approximately 1.6% of births). This was done as twin pregnancies face a very high mortality risk (4-6-fold increase). In addition, the analysis is complicated by the clustering of birth on one mother, why most epidemiological studies exclude multiples.
Replication	We have included the analysis files.
Randomization	no randomization for this study
Blinding	no blinding

Reporting for specific materials, systems and methods

We require information from authors about some types of materials, experimental systems and methods used in many studies. Here, indicate whether each material, system or method listed is relevant to your study. If you are not sure if a list item applies to your research, read the appropriate section before selecting a response.

Materials & experimental systems

n/a	Involved in the study
☒	☐ Antibodies
☒	☐ Eukaryotic cell lines
☒	☐ Palaeontology and archaeology
☒	☐ Animals and other organisms
☐	☒ Clinical data
☒	☐ Dual use research of concern
☒	☐ Plants

Methods

n/a	Involved in the study
☒	☐ ChIP-seq
☒	☐ Flow cytometry
☒	☐ MRI-based neuroimaging

Clinical data

Policy information about clinical studies

All manuscripts should comply with the ICMJE guidelines for publication of clinical research and a completed CONSORT checklist must be included with all submissions.

Clinical trial registration

ALERT is registered (June 17, 2020) in the Pan African Clinical Trial Registry at 202006793783148

Study protocol

https://bmchealthservres.biomedcentral.com/articles/10.1186/s12913-021-07155-z

Data collection

This study included singleton births from a prospective observational study in 16 hospitals in Benin, Malawi, Tanzania and Uganda collected as part of the Action Leveraging Evidence to Reduce perinatal morTality and morbidity (ALERT) study.50 Benin, Malawi, Tanzania and Uganda are low- and lower middle-income countries, facing a large perinatal mortality burden.1 The countries are categorised as low or lower-middle income countries with Real Gross Domestic Product per capita of between 1,500 (Malawi) to 3,300 (Benin) US dollar (Supplementary Table S1). Uptake to antenatal and childbirth care in facilities is improving but health systems are weak. Childbirth care faces larger out-of-pocket expenses, particular in Benin and Uganda, restricting preventive uptake of hospital care.

In each of the countries, four medium-size hospitals were included with more than 2500 births per annum. Hospitals were typically district or regional public and private-for-nonprofit (faith-based) facilities, although the national referral hospital in Cotonou, Benin also took part in the study. In this analysis, we included all mother-baby pairs admitted for childbirth in any of the hospitals between 1st July 2021 and 31th December 2023. We excluded mother-baby pairs who were referred to the hospitals after giving birth.

Outcomes

Our main outcomes were stillbirths (including both ante- and intrapartum deaths) and very early neonatal deaths documented in REDCap by nurse-midwives in each hospital. Stillbirths were defined as the death of a foetus before birth, weighing at least 1000 grams, and which could not be resuscitated after birth. Perinatal deaths included both stillbirths and deaths within the first 24 hours (very early neonatal deaths). We chose 24 hours as a cut-off as mothers were usually discharged the day after birth.

Plants

Seed stocks

Report on the source of all seed stocks or other plant material used. If applicable, state the seed stock centre and catalogue number. If plant specimens were collected from the field, describe the collection location, date and sampling procedures.

Novel plant genotypes

Describe the methods by which all novel plant genotypes were produced. This includes those generated by transgenic approaches, gene editing, chemical/radiation-based mutagenesis and hybridization. For transgenic lines, describe the transformation method, the number of independent lines analyzed and the generation upon which experiments were performed. For gene-edited lines, describe the editor used, the endogenous sequence targeted for editing, the targeting guide RNA sequence (if applicable) and how the editor was applied.

Authentication

Describe any authentication procedures for each seed stock used or novel genotype generated. Describe any experiments used to assess the effect of a mutation and, where applicable, how potential secondary effects (e.g. second site T-DNA insertions, mosiacism, off-target gene editing) were examined.
