# Supplementary material for: A time-stratified, case–crossover study of heat exposure and perinatal mortality from 16 hospitals in sub-Saharan Africa
Source: Nat Med. 2024 Sep 3;30(11):3106–13. doi: 10.1038/s41591-024-03245-7 (PMC11564089; doi:10.1038/s41591-024-03245-7)
Supplement: Supplementary file 3 — Analysis codes—descriptive analysis. [file 41591_2024_3245_MOESM3_ESM.pdf]

--------Summary descriptives table by 'country'---------

	[ALL] N=138015	Benin N=26390	Malawi N=50491	Tanzania N=23811	Uganda N=37323
all_still:					
No	133121 (96.5%)	24496 (92.8%)	49547 (98.1%)	23442 (98.5%)	35636 (95.5%)
Yes	4886 (3.5%)	1893 (7.2%)	944 (1.9%)	369 (1.5%)	1680 (4.5%)
intra_still:					
No	135273 (98.0%)	25288 (95.8%)	50022 (99.1%)	23652 (99.3%)	36311 (97.3%)
Yes	2734 (2.0%)	1101 (4.2%)	469 (0.9%)	159 (0.7%)	1005 (2.7%)
ante_still:					
No	135855 (98.4%)	25597 (97.0%)	50016 (99.1%)	23601 (99.1%)	36641 (98.2%)
Yes	2152 (1.6%)	792 (3.0%)	475 (0.9%)	210 (0.9%)	675 (1.8%)
per_mort:					
No	132135 (95.7%)	24280 (92.0%)	49206 (97.5%)	23342 (98.0%)	35307 (94.6%)
Yes	5880 (4.3%)	2110 (8.0%)	1285 (2.5%)	469 (2.0%)	2016 (5.4%)
mat_age:					
<35	122371 (88.8%)	22435 (85.1%)	45939 (91.2%)	20019 (84.1%)	33978 (91.0%)
>=35	15499 (11.2%)	3943 (14.9%)	4424 (8.8%)	3792 (15.9%)	3340 (9.0%)
referred:					
No	112454 (81.7%)	12190 (46.2%)	45859 (90.8%)	22677 (95.2%)	31728 (85.8%)
Yes	25199 (18.3%)	14191 (53.8%)	4629 (9.2%)	1133 (4.8%)	5246 (14.2%)
q11hiv:					
No	120899 (96.9%)	21257 (98.4%)	42254 (96.2%)	23028 (96.9%)	34360 (96.6%)
Yes	3932 (3.1%)	353 (1.6%)	1658 (3.8%)	730 (3.1%)	1191 (3.4%)
ht:					
No	129129 (93.6%)	21345 (80.9%)	49614 (98.3%)	22682 (95.3%)	35488 (95.1%)
Yes	8853 (6.4%)	5032 (19.1%)	877 (1.7%)	1129 (4.7%)	1815 (4.9%)
preg_num:					
1st	51212 (37.1%)	7264 (27.6%)	22914 (45.4%)	8485	

```
(35.6%)  12549 (33.6%)
    2 or +      86727 (62.9%)  19100 (72.4%) 27542 (54.6%) 15325
(64.4%) 24760 (66.4%)
q29sex:
    Girl        67448 (48.9%)  12506 (47.4%) 24442 (48.4%) 11783
(49.5%) 18717 (50.2%)
    Boy         70516 (51.1%)  13866 (52.6%) 26033 (51.6%) 12022
(50.5%) 18595 (49.8%)
preterm:
    No          118282 (87.5%) 21676 (83.0%) 44511 (89.4%) 20568
(86.5%) 31527 (88.9%)
    Yes         16848 (12.5%)  4432 (17.0%)  5284 (10.6%)  3197
(13.5%)  3935 (11.1%)
low_bw:
    No          120246 (87.4%) 21298 (81.0%) 44052 (87.6%) 21151
(88.9%) 33745 (90.8%)
    Yes         17311 (12.6%)  4982 (19.0%)  6262 (12.4%)  2652
(11.1%)   3415 (9.2%)
prol_obst_lab:
    No          127258 (92.2%) 24773 (93.9%) 46281 (91.7%) 21421
(90.0%) 34783 (93.2%)
    Yes          10757 (7.8%)   1617 (6.1%)   4210 (8.3%)  2390
(10.0%)   2540 (6.8%)
deliv_mode:
    Spontaneous 98488 (71.4%)  13578 (51.5%) 41171 (81.5%) 16887
(70.9%) 26852 (72.0%)
    Caesarean   38143 (27.6%)  12477 (47.3%) 8561 (17.0%)  6758
(28.4%)  10347 (27.7%)
    Others       1376 (1.0%)    332 (1.3%)    759 (1.5%)    165
(0.7%)    120 (0.3%)
aph:
    No          136092 (98.6%) 25650 (97.2%) 50141 (99.3%) 23641
(99.3%) 36660 (98.2%)
    Yes          1923 (1.4%)    740 (2.8%)    350 (0.7%)    170
(0.7%)    663 (1.8%)
temp_week_9       22.8 (3.3)    26.9 (1.2)    20.1 (2.9)    24.8
(1.6)    22.1 (0.8)
temp_week_min_9   19.0 (4.1)    25.0 (1.1)    15.3 (3.1)    20.3
(2.0)    18.8 (0.8)
temp_max_week_9   27.1 (3.2)    29.8 (1.8)    25.2 (3.0)    30.2
(2.2)    25.9 (1.4)
---------------------------------------------------------------------
------------------
```
