# Supplementary material for: A time-stratified, case–crossover study of heat exposure and perinatal mortality from 16 hospitals in sub-Saharan Africa
Source: Nat Med. 2024 Sep 3;30(11):3106–13. doi: 10.1038/s41591-024-03245-7 (PMC11564089; doi:10.1038/s41591-024-03245-7)
Supplement: Supplementary file 4 — Analysis codes—results. [file 41591_2024_3245_MOESM4_ESM.pdf]

```
          Country Exposure Temp 75th       Stillbirths Temp 75th
Antepartum Temp 75th        Intrapartum
 1:          Benin    tmean      28.0 1.14 (0.65; 2.01)      27.9
1.53 (0.60; 3.88)      28.0  0.71 (0.34; 1.48)
 2:         Malawi    tmean      22.1 1.66 (0.83; 3.33)      22.2
1.10 (0.40; 3.03)      22.0  2.81 (1.06; 7.41)
 3:       Tanzania    tmean      25.9 0.94 (0.30; 2.92)      25.9
0.40 (0.07; 2.15)      25.8 9.03 (1.01; 80.42)
 4:         Uganda    tmean      22.7 1.32 (0.82; 2.15)      22.8
1.31 (0.58; 2.97)      22.7  1.39 (0.74; 2.61)
 5: Meta-analysis    tmean        NA 1.29 (0.95; 1.77)        NA
1.18 (0.71; 1.95)        NA  1.64 (0.74; 3.63)
 6:          Benin     tmax      31.4 1.07 (0.67; 1.71)      31.3
1.01 (0.52; 1.99)      31.4  1.05 (0.55; 2.00)
 7:         Malawi     tmax      27.7 2.10 (0.97; 4.55)      27.8
1.87 (0.60; 5.84)      27.6  3.02 (0.98; 9.25)
 8:       Tanzania     tmax      31.9 0.57 (0.14; 2.32)      31.9
0.18 (0.02; 1.66)      31.7 3.72 (0.41; 33.88)
 9:         Uganda     tmax      26.8 1.24 (0.70; 2.21)      26.8
1.35 (0.53; 3.47)      26.8  1.01 (0.46; 2.19)
10: Meta-analysis     tmax        NA 1.22 (0.89; 1.68)        NA
1.12 (0.69; 1.82)        NA  1.33 (0.81; 2.20)
11:          Benin     tmin      25.9 1.36 (0.68; 2.69)      25.8
1.64 (0.55; 4.89)      26.0  1.23 (0.52; 2.87)
12:         Malawi     tmin      17.9 1.27 (0.59; 2.74)      18.0
0.97 (0.32; 2.94)      17.8  1.56 (0.54; 4.47)
13:       Tanzania     tmin      22.0 1.37 (0.37; 5.11)      22.1
0.69 (0.11; 4.34)      22.0 6.24 (0.52; 75.35)
14:         Uganda     tmin      19.4 1.26 (0.62; 2.57)      19.4
0.49 (0.16; 1.57)      19.4  2.33 (0.97; 5.59)
15: Meta-analysis     tmin        NA 1.30 (0.88; 1.94)        NA
0.91 (0.50; 1.68)        NA  1.74 (1.04; 2.92)
    Temp 75th  Perinatal deaths
 1:      28.0 1.30 (0.76; 2.20)
 2:      22.2 1.39 (0.76; 2.52)
 3:      25.9 1.03 (0.39; 2.69)
 4:      22.7 1.42 (0.91; 2.22)
 5:        NA 1.34 (1.01; 1.78)
 6:      31.3 1.10 (0.71; 1.70)
 7:      27.8 1.83 (0.94; 3.59)
 8:      31.8 0.56 (0.16; 1.93)
 9:      26.9 1.35 (0.79; 2.28)
10:        NA 1.24 (0.93; 1.66)
11:      25.9 1.57 (0.85; 2.91)
12:      17.9 1.07 (0.51; 2.25)
13:      22.1 1.31 (0.38; 4.53)
14:      19.4 1.21 (0.62; 2.36)
15:        NA 1.30 (0.90; 1.88)
          Country Exposure Temp 50th       Stillbirths Temp 50th
Antepartum Temp 50th        Intrapartum
 1:          Benin    tmean      27.1 0.95 (0.53; 1.71)      27.0
1.20 (0.45; 3.16)      27.2  0.63 (0.30; 1.34)
 2:         Malawi    tmean      20.1 1.34 (0.65; 2.74)      20.2
1.05 (0.37; 3.00)      20.0  1.81 (0.66; 5.00)
```

```
 3:      Tanzania    tmean      24.6 1.04 (0.35; 3.09)      24.6
0.38 (0.07; 1.89)      24.7 9.54 (1.17; 77.72)
 4:        Uganda    tmean      22.0 1.36 (0.84; 2.22)      22.0
1.31 (0.58; 2.97)      21.9  1.42 (0.75; 2.69)
 5: Meta-analysis    tmean        NA 1.19 (0.87; 1.64)        NA
1.07 (0.65; 1.79)        NA  1.41 (0.67; 2.96)
 6:         Benin     tmax      29.9 1.23 (0.71; 2.12)      29.8
1.03 (0.46; 2.31)      30.0  1.29 (0.61; 2.74)
 7:        Malawi     tmax      24.9 1.69 (0.77; 3.70)      25.1
1.65 (0.53; 5.19)      24.8  2.04 (0.66; 6.28)
 8:      Tanzania     tmax      29.6 0.53 (0.14; 2.04)      29.6
0.17 (0.02; 1.32)      29.6 3.34 (0.40; 28.04)
 9:        Uganda     tmax      25.7 1.27 (0.70; 2.29)      25.8
1.44 (0.56; 3.75)      25.7  0.99 (0.45; 2.18)
10: Meta-analysis     tmax        NA 1.25 (0.88; 1.76)        NA
1.12 (0.66; 1.90)        NA  1.33 (0.83; 2.15)
11:         Benin     tmin      25.0 1.20 (0.62; 2.33)      24.8
1.49 (0.51; 4.36)      25.1  1.08 (0.47; 2.45)
12:        Malawi     tmin      15.9 1.10 (0.52; 2.35)      16.0
0.91 (0.31; 2.68)      15.8  1.26 (0.44; 3.60)
13:      Tanzania     tmin      20.9 1.34 (0.36; 5.03)      20.9
0.66 (0.11; 4.13)      20.9 4.63 (0.44; 48.71)
14:        Uganda     tmin      18.8 1.30 (0.66; 2.55)      18.8
0.58 (0.19; 1.73)      18.8  2.20 (0.96; 5.04)
15: Meta-analysis     tmin        NA 1.22 (0.83; 1.78)        NA
0.90 (0.50; 1.62)        NA  1.54 (0.94; 2.54)
    Temp 50th  Perinatal deaths
 1:      27.1 1.09 (0.63; 1.89)
 2:      20.1 1.11 (0.60; 2.07)
 3:      24.7 1.06 (0.42; 2.67)
 4:      22.0 1.46 (0.93; 2.30)
 5:        NA 1.23 (0.92; 1.64)
 6:      29.9 1.20 (0.72; 2.02)
 7:      25.0 1.48 (0.75; 2.92)
 8:      29.6 0.54 (0.17; 1.75)
 9:      25.8 1.38 (0.80; 2.37)
10:        NA 1.24 (0.91; 1.71)
11:      25.0 1.39 (0.76; 2.55)
12:      15.8 0.93 (0.45; 1.89)
13:      21.0 1.21 (0.36; 4.06)
14:      18.8 1.25 (0.67; 2.35)
15:        NA 1.20 (0.84; 1.71)
        beta        se   i2                  model  cen          low
high   or or_low or_high
1: 0.2563587 0.1594405 0.00            Stillbirth 75th -0.056138846
0.5688563 1.29   0.95    1.77
2: 0.4920003 0.4066746 0.63 Intrapartum stillbirth 75th -0.305067208
1.2890678 1.64   0.74    3.63
3: 0.1665774 0.2565032 0.00  Antepartum stillbirth 75th -0.336159558
0.6693144 1.18   0.71    1.95
4: 0.2918684 0.1444793 0.00        Perinatal death 75th  0.008694206
0.5750426 1.34   1.01    1.78
5: 0.6479472 0.2701562 0.36            Stillbirth 75th  0.118450702
1.1774437 1.91   1.13    3.25
```

```
6: 1.3210822 0.8215560 0.75 Intrapartum stillbirth 75th -0.289137916
2.9313024 3.75   0.75   18.75
7: 0.5441817 0.3633788 0.35  Antepartum stillbirth 75th -0.168027587
1.2563910 1.72   0.85    3.51
8: 0.6709859 0.1982922 0.02        Perinatal death 75th  0.282340362
1.0596314 1.96   1.33    2.89
               hr_all           time
1:  1.29 (0.95; 1.77)     Whole year
2:  1.64 (0.74; 3.63)     Whole year
3:  1.18 (0.71; 1.95)     Whole year
4:  1.34 (1.01; 1.78)     Whole year
5:  1.91 (1.13; 3.25) Hottest months
6: 3.75 (0.75; 18.75) Hottest months
7:  1.72 (0.85; 3.51) Hottest months
8:  1.96 (1.33; 2.89) Hottest months
            beta         se   i2           model  cen          low
high   or or_low or_high
 1:  0.106156190 0.10135102 0.00   All stillbirth 75th -0.092488154
0.3048005 1.11   0.91    1.36
 2: -0.005482957 0.24728171 0.56 Intra stillbirth 75th -0.490146205
0.4791803 0.99   0.61    1.61
 3:  0.300370267 0.15274528 0.00      Antermortum 75th  0.000995015
0.5997455 1.35   1.00    1.82
 4:  0.160706811 0.09214322 0.00   Pernatal death 75th -0.019890590
0.3413042 1.17   0.98    1.41
 5:  0.161486225 0.11210408 0.00   All stillbirth 75th -0.058233725
0.3812062 1.18   0.94    1.46
 6:  0.055217422 0.28921809 0.62 Intra stillbirth 75th -0.511639616
0.6220745 1.06   0.60    1.86
 7:  0.402397327 0.17377204 0.00      Antermortum 75th  0.061810396
0.7429843 1.50   1.06    2.10
 8:  0.203454486 0.10159817 0.00   Pernatal death 75th  0.004325732
0.4025832 1.23   1.00    1.50
 9:  0.251837605 0.12160957 0.00   All stillbirth 75th  0.013487234
0.4901880 1.29   1.01    1.63
10:  0.119468110 0.21040610 0.37 Intra stillbirth 75th -0.292920261
0.5318565 1.13   0.75    1.70
11:  0.407209819 0.18944726 0.00      Antermortum 75th  0.035900018
0.7785196 1.50   1.04    2.18
12:  0.278730947 0.10877872 0.00   Pernatal death 75th  0.065528566
0.4919333 1.32   1.07    1.64
13:  0.256358735 0.15944047 0.00   All stillbirth 75th -0.056138846
0.5688563 1.29   0.95    1.77
14:  0.492000308 0.40667457 0.63 Intra stillbirth 75th -0.305067208
1.2890678 1.64   0.74    3.63
15:  0.166577408 0.25650317 0.00      Antermortum 75th -0.336159558
0.6693144 1.18   0.71    1.95
16:  0.291868389 0.14447928 0.00   Pernatal death 75th  0.008694206
0.5750426 1.34   1.01    1.78
               hr_all   lag             meas
 1: 1.11 (0.91; 1.36)  lag0 Mean temperature
 2: 0.99 (0.61; 1.61)  lag0 Mean temperature
 3: 1.35 (1.00; 1.82)  lag0 Mean temperature
 4: 1.17 (0.98; 1.41)  lag0 Mean temperature
```

```
 5: 1.18 (0.94; 1.46) lag01 Mean temperature
 6: 1.06 (0.60; 1.86) lag01 Mean temperature
 7: 1.50 (1.06; 2.10) lag01 Mean temperature
 8: 1.23 (1.00; 1.50) lag01 Mean temperature
 9: 1.29 (1.01; 1.63) lag02 Mean temperature
10: 1.13 (0.75; 1.70) lag02 Mean temperature
11: 1.50 (1.04; 2.18) lag02 Mean temperature
12: 1.32 (1.07; 1.64) lag02 Mean temperature
13: 1.29 (0.95; 1.77) lag06 Mean temperature
14: 1.64 (0.74; 3.63) lag06 Mean temperature
15: 1.18 (0.71; 1.95) lag06 Mean temperature
16: 1.34 (1.01; 1.78) lag06 Mean temperature
           beta         se   i2                  model          low
high   or or_low or_high
 1: 0.256358735 0.15944047 0.00            Stillbirth -0.056138846
0.5688563 1.29   0.95    1.77
 2: 0.492000308 0.40667457 0.63 Intrapartum stillbirth -0.305067208
1.2890678 1.64   0.74    3.63
 3: 0.166577408 0.25650317 0.00  Antepartum stillbirth -0.336159558
0.6693144 1.18   0.71    1.95
 4: 0.291868389 0.14447928 0.00        Perinatal death  0.008694206
0.5750426 1.34   1.01    1.78
 5: 0.034010500 0.10805934 0.00            Stillbirth -0.177781916
0.2458029 1.03   0.84    1.28
 6: 0.095208287 0.17615539 0.24 Intrapartum stillbirth -0.250049933
0.4404665 1.10   0.78    1.55
 7: 0.008162232 0.16913477 0.00  Antepartum stillbirth -0.323335831
0.3396603 1.01   0.72    1.40
 8: 0.050468343 0.09841252 0.00        Perinatal death -0.142416655
0.2433533 1.05   0.87    1.28
 9: 0.047324827 0.11721829 0.00            Stillbirth -0.182418809
0.2770685 1.05   0.83    1.32
10: 0.122240189 0.19705369 0.29 Intrapartum stillbirth -0.263977944
0.5084583 1.13   0.77    1.66
11: 0.022146925 0.18399448 0.00  Antepartum stillbirth -0.338475622
0.3827695 1.02   0.71    1.47
12: 0.067776946 0.10691744 0.00        Perinatal death -0.141777392
0.2773313 1.07   0.87    1.32
13: 0.039735970 0.15621058 0.00            Stillbirth -0.266431141
0.3459031 1.04   0.77    1.41
14: 0.124708042 0.29415281 0.49 Intrapartum stillbirth -0.451820868
0.7012370 1.13   0.64    2.02
15: 0.056450113 0.22821954 0.00  Antepartum stillbirth -0.390851962
0.5037522 1.06   0.68    1.65
16: 0.086569152 0.14339456 0.00        Perinatal death -0.194479013
0.3676173 1.09   0.82    1.44
17: 0.127630901 0.15632689 0.00            Stillbirth -0.178764174
0.4340260 1.14   0.84    1.54
18: 0.248051567 0.30515969 0.45 Intrapartum stillbirth -0.350050438
0.8461536 1.28   0.70    2.33
19: 0.083241940 0.24389409 0.00  Antepartum stillbirth -0.394781686
0.5612656 1.09   0.67    1.75
20: 0.168119283 0.14181000 0.00        Perinatal death -0.109823210
0.4460618 1.18   0.90    1.56
```

```
21: 0.186028480 0.15801286 0.00              Stillbirth -0.123671032
0.4957280 1.20   0.88    1.64
22: 0.387881493 0.35901056 0.58 Intrapartum stillbirth -0.315766268
1.0915293 1.47   0.73    2.98
23: 0.105858344 0.24989276 0.00  Antepartum stillbirth -0.383922472
0.5956392 1.11   0.68    1.81
24: 0.222454970 0.14345668 0.00         Perinatal death -0.058714965
0.5036249 1.25   0.94    1.65
25: 0.264241301 0.15754265 0.00              Stillbirth -0.044536624
0.5730192 1.30   0.96    1.77
26: 0.516944812 0.40308425 0.63 Intrapartum stillbirth -0.273085795
1.3069754 1.68   0.76    3.69
27: 0.132612964 0.25319096 0.00  Antepartum stillbirth -0.363632201
0.6288581 1.14   0.70    1.88
28: 0.296579356 0.14235718 0.00         Perinatal death  0.017564406
0.5755943 1.35   1.02    1.78
29: 0.229055972 0.15987010 0.00              Stillbirth -0.084283661
0.5423956 1.26   0.92    1.72
30: 0.437366641 0.30586458 0.46 Intrapartum stillbirth -0.162116924
1.0368502 1.55   0.85    2.82
31: 0.126939706 0.25045326 0.00  Antepartum stillbirth -0.363939667
0.6178191 1.14   0.69    1.85
32: 0.255341487 0.14503391 0.00         Perinatal death -0.028919753
0.5396027 1.29   0.97    1.72
33: 0.253054119 0.15965226 0.00              Stillbirth -0.059858568
0.5659668 1.29   0.94    1.76
34: 0.508669640 0.36251729 0.58 Intrapartum stillbirth -0.201851194
1.2191905 1.66   0.82    3.38
35: 0.152643273 0.25363169 0.00  Antepartum stillbirth -0.344465700
0.6497522 1.16   0.71    1.92
36: 0.279918265 0.14485827 0.00         Perinatal death -0.003998724
0.5638353 1.32   1.00    1.76
           beta          se   i2                  model          low
high   or or_low or_high
               hr_all                sens
 1: 1.29 (0.95; 1.77)          Main model
 2: 1.64 (0.74; 3.63)          Main model
 3: 1.18 (0.71; 1.95)          Main model
 4: 1.34 (1.01; 1.78)          Main model
 5: 1.03 (0.84; 1.28)         1 knot 50th
 6: 1.10 (0.78; 1.55)         1 knot 50th
 7: 1.01 (0.72; 1.40)         1 knot 50th
 8: 1.05 (0.87; 1.28)         1 knot 50th
 9: 1.05 (0.83; 1.32)         1 knot 75th
10: 1.13 (0.77; 1.66)         1 knot 75th
11: 1.02 (0.71; 1.47)         1 knot 75th
12: 1.07 (0.87; 1.32)         1 knot 75th
13: 1.04 (0.77; 1.41)   2 knot 25 and 50th
14: 1.13 (0.64; 2.02)   2 knot 25 and 50th
15: 1.06 (0.68; 1.65)   2 knot 25 and 50th
16: 1.09 (0.82; 1.44)   2 knot 25 and 50th
17: 1.14 (0.84; 1.54)   2 knot 50 and 75th
18: 1.28 (0.70; 2.33)   2 knot 50 and 75th
19: 1.09 (0.67; 1.75)   2 knot 50 and 75th
```

```
20: 1.18 (0.90; 1.56)     2 knot 50 and 75th
21: 1.20 (0.88; 1.64)     2 knot 50 and 90th
22: 1.47 (0.73; 2.98)     2 knot 50 and 90th
23: 1.11 (0.68; 1.81)     2 knot 50 and 90th
24: 1.25 (0.94; 1.65)     2 knot 50 and 90th
25: 1.30 (0.96; 1.77)     2 knot 75 and 90th
26: 1.68 (0.76; 3.69)     2 knot 75 and 90th
27: 1.14 (0.70; 1.88)     2 knot 75 and 90th
28: 1.35 (1.02; 1.78)     2 knot 75 and 90th
29: 1.26 (0.92; 1.72) 3 knot 25, 50 and 75th
30: 1.55 (0.85; 2.82) 3 knot 25, 50 and 75th
31: 1.14 (0.69; 1.85) 3 knot 25, 50 and 75th
32: 1.29 (0.97; 1.72) 3 knot 25, 50 and 75th
33: 1.29 (0.94; 1.76) 3 knot 10, 50 and 90th
34: 1.66 (0.82; 3.38) 3 knot 10, 50 and 90th
35: 1.16 (0.71; 1.92) 3 knot 10, 50 and 90th
36: 1.32 (1.00; 1.76) 3 knot 10, 50 and 90th
               hr_all                   sens
          beta        se   i2                  model  cen
low      high   or or_low or_high
1:  0.25635874 0.1594405 0.00             Stillbirth 75th
-0.056138846 0.5688563 1.29   0.95    1.77
2:  0.49200031 0.4066746 0.63 Intrapartum stillbirth 75th
-0.305067208 1.2890678 1.64   0.74    3.63
3:  0.16657741 0.2565032 0.00  Antepartum stillbirth 75th
-0.336159558 0.6693144 1.18   0.71    1.95
4:  0.29186839 0.1444793 0.00        Perinatal death 75th
0.008694206 0.5750426 1.34   1.01    1.78
5:  0.04140698 0.3842313 0.57             Stillbirth 75th
-0.711672607 0.7944866 1.04   0.49    2.21
6:  0.50730666 0.9577834 0.66 Intrapartum stillbirth 75th
-1.369914300 2.3845276 1.66   0.25   10.85
7: -0.03046354 0.4570420 0.36  Antepartum stillbirth 75th
-0.926249360 0.8653223 0.97   0.40    2.38
8:  0.27476082 0.2333967 0.03        Perinatal death 75th
-0.182688335 0.7322100 1.32   0.83    2.08
               hr_all                     exp
1:  1.29 (0.95; 1.77)              Main model
2:  1.64 (0.74; 3.63)              Main model
3:  1.18 (0.71; 1.95)              Main model
4:  1.34 (1.01; 1.78)              Main model
5:  1.04 (0.49; 2.21) Without referral births
6: 1.66 (0.25; 10.85) Without referral births
7:  0.97 (0.40; 2.38) Without referral births
8:  1.32 (0.83; 2.08) Without referral births
        beta        se   i2                  model          low
high   or or_low or_high            hr_all
1: 0.2563587 0.1594405 0.00             Stillbirth -0.056138846
0.5688563 1.29   0.95    1.77 1.29 (0.95; 1.77)
2: 0.4920003 0.4066746 0.63 Intrapartum stillbirth -0.305067208
1.2890678 1.64   0.74    3.63 1.64 (0.74; 3.63)
3: 0.1665774 0.2565032 0.00  Antepartum stillbirth -0.336159558
0.6693144 1.18   0.71    1.95 1.18 (0.71; 1.95)
4: 0.2918684 0.1444793 0.00        Perinatal death  0.008694206
```

```
0.5750426 1.34   1.01    1.78 1.34 (1.01; 1.78)
5: 0.2982135 0.1828654 0.11            Stillbirth -0.060196008
0.6566230 1.35   0.94    1.93 1.35 (0.94; 1.93)
6: 0.5089230 0.4750557 0.66 Intrapartum stillbirth -0.422169009
1.4400150 1.66   0.66    4.22 1.66 (0.66; 4.22)
7: 0.3011163 0.2909557 0.23  Antepartum stillbirth -0.269146374
0.8713789 1.35   0.76    2.39 1.35 (0.76; 2.39)
8: 0.7455280 0.4285438 0.00                  <NA> -0.094402486
1.5854584 2.11   0.91    4.88 2.11 (0.91; 4.88)
9: 0.3881615 0.1660860 0.09       Perinatal death  0.062638841
0.7136841 1.47   1.06    2.04 1.47 (1.06; 2.04)
                exp        meas
1: Mean temperature Not adj. RH
2: Mean temperature Not adj. RH
3: Mean temperature Not adj. RH
4: Mean temperature Not adj. RH
5: Mean temperature     adj. RH
6: Mean temperature     adj. RH
7: Mean temperature     adj. RH
8: Mean temperature     adj. RH
9: Mean temperature     adj. RH
     exp  cen resolution    All stillbirth       Antermortum  Intra
stillbirth    Pernatal death
1: tmean 75th    28x28km 1.28 (0.92; 1.79) 1.19 (0.71; 2.00) 1.56
(0.74; 3.30) 1.24 (0.92; 1.69)
2: tmean 75th      9x9km 1.29 (0.95; 1.77) 1.18 (0.71; 1.95) 1.64
(0.74; 3.63) 1.34 (1.01; 1.78)
3:  tmax 75th    28x28km 1.32 (0.89; 1.97) 1.12 (0.62; 2.03) 2.01
(0.78; 5.20) 1.33 (0.93; 1.88)
4:  tmax 75th      9x9km 1.22 (0.89; 1.68) 1.12 (0.69; 1.82) 1.33
(0.81; 2.20) 1.24 (0.93; 1.66)
5:  tmin 75th    28x28km 1.31 (0.87; 1.98) 1.14 (0.61; 2.12) 1.49
(0.85; 2.62) 1.16 (0.80; 1.68)
6:  tmin 75th      9x9km 1.30 (0.88; 1.94) 0.91 (0.50; 1.68) 1.74
(1.04; 2.92) 1.30 (0.90; 1.88)
    model                      out country              Mean
Min                 Max
 1:   Cox        Perinatal death   First 0.87 (0.55; 1.35) 0.81
(0.56; 1.17) 1.05 (0.66; 1.67)
 2:   Cox        Perinatal death  Second 1.11 (0.76; 1.60) 1.15
(0.86; 1.53) 1.14 (0.64; 2.04)
 3:   Cox        Perinatal death   Third 2.03 (1.67; 2.48) 1.48
(0.86; 2.53) 1.61 (0.92; 2.80)
 4:   Cox Intrapartum stillbirths   First 0.63 (0.34; 1.16) 0.90
(0.42; 1.92) 0.80 (0.36; 1.80)
 5:   Cox Intrapartum stillbirths  Second 0.95 (0.44; 2.04) 1.29
(0.95; 1.75) 1.02 (0.37; 2.77)
 6:   Cox Intrapartum stillbirths   Third 1.52 (0.93; 2.48) 1.23
(0.59; 2.55) 1.25 (0.66; 2.36)
 7:   Cox  Antepartum stillbirths   First 1.04 (0.72; 1.51) 0.59
(0.45; 0.77) 1.21 (0.84; 1.75)
 8:   Cox  Antepartum stillbirths  Second 0.96 (0.74; 1.25) 0.95
(0.72; 1.26) 1.00 (0.73; 1.38)
 9:   Cox  Antepartum stillbirths   Third 2.22 (1.85; 2.66) 1.86
```

```
(1.16; 2.97) 1.88 (1.05; 3.34)
10:   Cox            Stillbirth   First 0.79 (0.50; 1.25) 0.80
(0.52; 1.22) 1.01 (0.59; 1.76)
11:   Cox            Stillbirth  Second 1.04 (0.65; 1.65) 1.10
(0.80; 1.51) 1.11 (0.57; 2.14)
12:   Cox            Stillbirth   Third 2.06 (1.70; 2.50) 1.50
(0.84; 2.66) 1.58 (0.94; 2.66)
           beta        se   i2                  model          low
high                 or or_low
 1:   0.38528751  0.2345277 0.28             Stillbirth  -0.07437837
0.8449534             1.47   0.93
 2:   0.76217091  0.5847725 0.58 Intrapartum stillbirth  -0.38396216
1.9083040             2.14   0.68
 3:  -0.12881448  0.3949203 0.00  Antepartum stillbirth  -0.90284412
0.6452152             0.88   0.41
 4:   0.43154551  0.2130242 0.00        Perinatal death   0.01402568
0.8490653             1.54   1.01
 5:   0.15147386  0.2366900 0.00             Stillbirth  -0.31242998
0.6153777             1.16   0.73
 6:  -0.10743988  0.3405917 0.12 Intrapartum stillbirth  -0.77498731
0.5601075             0.90   0.46
 7:   0.48194745  0.3839801 0.00  Antepartum stillbirth  -0.27063964
1.2345345             1.62   0.76
 8:   0.19848126  0.2109552 0.00        Perinatal death  -0.21498327
0.6119458             1.22   0.81
 9:  -0.02009406  0.3302998 0.21             Stillbirth  -0.66746985
0.6272817             0.98   0.51
10:  -0.04686585  0.7593410 0.55 Intrapartum stillbirth  -1.53514681
1.4414151             0.95   0.22
11:  -0.11242254  1.1171025 0.67  Antepartum stillbirth  -2.30190314
2.0770581             0.89   0.10
12:  -0.04982823  0.2801217 0.00        Perinatal death  -0.59885677
0.4992003             0.95   0.55
13:   0.35035074  0.2050772 0.00             Stillbirth  -0.05159310
0.7522946             1.42   0.95
14:   0.44696829  0.3463714 0.55 Intrapartum stillbirth  -0.23190719
1.1258438             1.56   0.79
15:   0.23787492  0.3231968 0.00  Antepartum stillbirth  -0.39557907
0.8713289             1.27   0.67
16:   0.32599941  0.1898788 0.00        Perinatal death  -0.04615615
0.6981550             1.39   0.95
17:   0.44845289  0.3052731 0.00             Stillbirth  -0.14987134
1.0467771             1.57   0.86
18:   0.55044734  0.4865173 0.31 Intrapartum stillbirth  -0.40310912
1.5040038             1.73   0.67
19:   0.30601169  0.4571370 0.00  Antepartum stillbirth  -0.58996046
1.2019838             1.36   0.55
20:   0.36940843  0.2784450 0.00        Perinatal death  -0.17633369
0.9151505             1.45   0.84
21:   0.15902720  0.2204745 0.00             Stillbirth  -0.27309493
0.5911493             1.17   0.76
22:   0.35613850  0.2971562 0.00 Intrapartum stillbirth  -0.22627702
0.9385540             1.43   0.80
23:   0.05454125  0.3752460 0.00  Antepartum stillbirth  -0.68092731
```

```
0.7900098                1.06   0.51
24:   0.12671785  0.1992934 0.00        Perinatal death  -0.26389000
0.5173257                1.14   0.77
25:   0.27426974  0.1848649 0.00             Stillbirth  -0.08805885
0.6365983                1.32   0.92
26:   0.25838727  0.3860722 0.47 Intrapartum stillbirth  -0.49830033
1.0150749                1.29   0.61
27:   0.27154049  0.4306500 0.51  Antepartum stillbirth  -0.57251795
1.1155989                1.31   0.56
28:   0.22907223  0.1687692 0.00        Perinatal death  -0.10170934
0.5598538                1.26   0.90
29:  -0.05880619  0.5141086 0.08             Stillbirth  -1.06644048
0.9488281                0.94   0.34
30:   0.13338699  0.7139729 0.48 Intrapartum stillbirth  -1.26597414
1.5327481                1.14   0.28
31:  -0.91192462  1.0310109 0.21  Antepartum stillbirth  -2.93266884
1.1088196                0.40   0.05
32:  -0.01221996  0.4947381 0.13        Perinatal death  -0.98188877
0.9574488                0.99   0.37
33:   0.10757374  0.2187639 0.00             Stillbirth  -0.32119568
0.5363432                1.11   0.73
34:   0.31017982  0.2865530 0.00 Intrapartum stillbirth  -0.25145373
0.8718134                1.36   0.78
35:  -0.12481928  0.3872471 0.00  Antepartum stillbirth  -0.88380968
0.6341711                0.88   0.41
36:   0.11085195  0.1988185 0.00        Perinatal death  -0.27882508
0.5005290                1.12   0.76
37:   0.32876594  0.2724860 0.00             Stillbirth  -0.20529689
0.8628288                1.39   0.81
38:   0.79253124  0.8748433 0.64 Intrapartum stillbirth  -0.92213003
2.5071925                2.21   0.40
39:   0.19563408  0.4044370 0.00  Antepartum stillbirth  -0.59704783
0.9883160                1.22   0.55
40:   0.36048746  0.2514400 0.00        Perinatal death  -0.13232592
0.8533008                1.43   0.88
41:   0.24998296  0.1863416 0.00             Stillbirth  -0.11523980
0.6152057                1.28   0.89
42:   0.31786609  0.3631581 0.51 Intrapartum stillbirth  -0.39391066
1.0296428                1.37   0.67
43:   0.22981912  0.3280079 0.30  Antepartum stillbirth  -0.41306457
0.8727028                1.26   0.66
44:   0.20512214  0.1675777 0.00        Perinatal death  -0.12332421
0.5335685                1.23   0.88
45:   2.99941809  2.2207075 0.00             Stillbirth  -1.35308864
7.3519248               20.07   0.26
46:  34.99858608 53.3375875 0.46 Intrapartum stillbirth -69.54116446
139.5383366 1583772542650948.75   0.00
47:   3.38504639  7.4885950 0.00  Antepartum stillbirth -11.29233019
18.0624230              29.52   0.00
48:   1.82389933  1.4666984 0.00        Perinatal death  -1.05077662
4.6985753                6.20   0.35
49:   0.25460061  0.1782384 0.00             Stillbirth  -0.09474019
0.6039414                1.29   0.91
50:   0.59024497  0.5111892 0.68 Intrapartum stillbirth  -0.41166749
```

```
1.5921574                 1.80   0.66
51:   0.15230862  0.2702802 0.00  Antepartum stillbirth  -0.37743084
0.6820481                 1.16   0.69
52:   0.21336121  0.1601387 0.00        Perinatal death  -0.10050485
0.5272273                 1.24   0.90
53:  -0.64357673  1.5010002 0.36             Stillbirth  -3.58548311
2.2983296                 0.53   0.03
54:  -1.08630062  3.0621718 0.53 Intrapartum stillbirth  -7.08804703
4.9154458                 0.34   0.00
55: -13.06200000 18.5760000   NA  Antepartum stillbirth -49.47029098
23.3462910                 0.00   0.00
56:   0.01020290  0.7373332 0.00        Perinatal death  -1.43494368
1.4553495                 1.01   0.24
57:   0.18513393  0.1807993 0.00             Stillbirth  -0.16922618
0.5394940                 1.20   0.84
58:   0.49232522  0.4777862 0.65 Intrapartum stillbirth  -0.44411859
1.4287690                 1.64   0.64
59:   0.08776782  0.2815622 0.00  Antepartum stillbirth  -0.46408393
0.6396196                 1.09   0.63
60:   0.18507037  0.1634366 0.00        Perinatal death  -0.13525953
0.5054003                 1.20   0.87
61:   0.54911219  1.0173226 0.36             Stillbirth  -1.44480342
2.5430278                 1.73   0.24
62:   0.44764539  0.9047141 0.00 Intrapartum stillbirth  -1.32556159
2.2208524                 1.56   0.27
63:   4.28821362  5.0121627 0.23  Antepartum stillbirth  -5.53544473
14.1118720                72.84   0.00
64:   0.36044746  0.9675032 0.36        Perinatal death  -1.53582393
2.2567189                 1.43   0.22
65:   0.16203649  0.1844416 0.00             Stillbirth  -0.19946249
0.5235355                 1.18   0.82
66:   0.37392277  0.3657643 0.54 Intrapartum stillbirth  -0.34296211
1.0908076                 1.45   0.71
67:   0.02883586  0.3096061 0.55  Antepartum stillbirth  -0.57798087
0.6356526                 1.03   0.56
68:   0.13439947  0.1678084 0.00        Perinatal death  -0.19449888
0.4632978                 1.14   0.82
69:   0.61154478  0.4454890 0.00             Stillbirth  -0.26159758
1.4846871                 1.84   0.77
70:   0.23009840  0.8080689 0.00 Intrapartum stillbirth  -1.35368755
1.8138844                 1.26   0.26
71:   0.67811906  0.9567516 0.31  Antepartum stillbirth  -1.19707964
2.5533178                 1.97   0.30
72:   0.61610472  0.4071655 0.00        Perinatal death  -0.18192501
1.4141345                 1.85   0.83
            beta         se   i2                  model          low
high                    or or_low
                                                             or_high
 1:                                                             2.33
 2:                                                             6.74
 3:                                                             1.91
 4:                                                             2.34
 5:                                                             1.85
 6:                                                             1.75
```

```
 7:                                                           3.44
 8:                                                           1.84
 9:                                                           1.87
10:                                                           4.23
11:                                                           7.98
12:                                                           1.65
13:                                                           2.12
14:                                                           3.08
15:                                                           2.39
16:                                                           2.01
17:                                                           2.85
18:                                                           4.50
19:                                                           3.33
20:                                                           2.50
21:                                                           1.81
22:                                                           2.56
23:                                                           2.20
24:                                                           1.68
25:                                                           1.89
26:                                                           2.76
27:                                                           3.05
28:                                                           1.75
29:                                                           2.58
30:                                                           4.63
31:                                                           3.03
32:                                                           2.61
33:                                                           1.71
34:                                                           2.39
35:                                                           1.89
36:                                                           1.65
37:                                                           2.37
38:                                                          12.27
39:                                                           2.69
40:                                                           2.35
41:                                                           1.85
42:                                                           2.80
43:                                                           2.39
44:                                                           1.71
45:                                                        1559.19
46: 398776549348943744569325375024654875175700437336544398802944 0.00
47:                                                    69889289.81
48:                                                         109.79
49:                                                           1.83
50:                                                           4.91
51:                                                           1.98
52:                                                           1.69
53:                                                           9.96
54:                                                         136.38
55:                                                 13777338984.60
56:                                                           4.29
57:                                                           1.72
58:                                                           4.17
59:                                                           1.90
60:                                                           1.66
```

```
61:                                                             12.72
62:                                                              9.22
63:                                                        1344956.15
64:                                                              9.55
65:                                                              1.69
66:                                                              2.98
67:                                                              1.89
68:                                                              1.59
69:                                                              4.41
70:                                                              6.13
71:                                                             12.85
72:                                                              4.11
                                                              or_high

hr_all         eff
 1:
1.47 (0.93; 2.33)        Girl
 2:
2.14 (0.68; 6.74)        Girl
 3:
0.88 (0.41; 1.91)        Girl
 4:
1.54 (1.01; 2.34)        Girl
 5:
1.16 (0.73; 1.85)         Boy
 6:
0.90 (0.46; 1.75)         Boy
 7:
1.62 (0.76; 3.44)         Boy
 8:
1.22 (0.81; 1.84)         Boy
 9:
0.98 (0.51; 1.87)         1st
10:
0.95 (0.22; 4.23)         1st
11:
0.89 (0.10; 7.98)         1st
12:
0.95 (0.55; 1.65)         1st
13:
1.42 (0.95; 2.12) 2nd or more
14:
1.56 (0.79; 3.08) 2nd or more
15:
1.27 (0.67; 2.39) 2nd or more
16:
1.39 (0.95; 2.01) 2nd or more
17:
1.57 (0.86; 2.85)     Preterm
18:
1.73 (0.67; 4.50)     Preterm
19:
1.36 (0.55; 3.33)     Preterm
20:
```

```
1.45 (0.84; 2.50)     Preterm
21:
1.17 (0.76; 1.81)     To term
22:
1.43 (0.80; 2.56)     To term
23:
1.06 (0.51; 2.20)     To term
24:
1.14 (0.77; 1.68)     To term
25:
1.32 (0.92; 1.89)   <35 years
26:
1.29 (0.61; 2.76)   <35 years
27:
1.31 (0.56; 3.05)   <35 years
28:
1.26 (0.90; 1.75)   <35 years
29:
0.94 (0.34; 2.58)  >=35 years
30:
1.14 (0.28; 4.63)  >=35 years
31:
0.40 (0.05; 3.03)  >=35 years
32:
0.99 (0.37; 2.61)  >=35 years
33:
1.11 (0.73; 1.71)    <2500 gr
34:
1.36 (0.78; 2.39)    <2500 gr
35:
0.88 (0.41; 1.89)    <2500 gr
36:
1.12 (0.76; 1.65)    <2500 gr
37:
1.39 (0.81; 2.37)  >= 2500 gr
38:
2.21 (0.40; 12.27)  >= 2500 gr
39:
1.22 (0.55; 2.69)  >= 2500 gr
40:
1.43 (0.88; 2.35)  >= 2500 gr
41:
1.28 (0.89; 1.85)    Negative
42:
1.37 (0.67; 2.80)    Negative
43:
1.26 (0.66; 2.39)    Negative
44:
1.23 (0.88; 1.71)    Negative
45:
20.07 (0.26; 1559.19)    Positive
46: 1583772542650948.75 (0.00;
3987765493489437445693253750246548751757004373365443988029440.00)
Positive
```

47:
29.52 (0.00; 69889289.81)   Positive
48:
6.20 (0.35; 109.79)   Positive
49:
1.29 (0.91; 1.83)   Normal
50:
1.80 (0.66; 4.91)   Normal
51:
1.16 (0.69; 1.98)   Normal
52:
1.24 (0.90; 1.69)   Normal
53:
0.53 (0.03; 9.96) Prolongued
54:
0.34 (0.00; 136.38) Prolongued
55:
0.00 (0.00; 13777338984.60) Prolongued
56:
1.01 (0.24; 4.29) Prolongued
57:
1.20 (0.84; 1.72)   No
58:
1.64 (0.64; 4.17)   No
59:
1.09 (0.63; 1.90)   No
60:
1.20 (0.87; 1.66)   No
61:
1.73 (0.24; 12.72)   Yes
62:
1.56 (0.27; 9.22)   Yes
63:
72.84 (0.00; 1344956.15)   Yes
64:
1.43 (0.22; 9.55)   Yes
65:
1.18 (0.82; 1.69)   No
66:
1.45 (0.71; 2.98)   No
67:
1.03 (0.56; 1.89)   No
68:
1.14 (0.82; 1.59)   No
69:
1.84 (0.77; 4.41)   Yes
70:
1.26 (0.26; 6.13)   Yes
71:
1.97 (0.30; 12.85)   Yes
72:
1.85 (0.83; 4.11)   Yes

hr_all   eff

	eff_mod
1:	Sex
2:	Sex
3:	Sex
4:	Sex
5:	Sex
6:	Sex
7:	Sex
8:	Sex
9:	N° pregnancies
10:	N° pregnancies
11:	N° pregnancies
12:	N° pregnancies
13:	N° pregnancies
14:	N° pregnancies
15:	N° pregnancies
16:	N° pregnancies
17:	Preterm
18:	Preterm
19:	Preterm
20:	Preterm
21:	Preterm
22:	Preterm
23:	Preterm
24:	Preterm
25:	Maternal age
26:	Maternal age
27:	Maternal age
28:	Maternal age
29:	Maternal age
30:	Maternal age
31:	Maternal age
32:	Maternal age
33:	Birthweight
34:	Birthweight
35:	Birthweight
36:	Birthweight
37:	Birthweight
38:	Birthweight
39:	Birthweight
40:	Birthweight
41:	HIV status
42:	HIV status
43:	HIV status
44:	HIV status
45:	HIV status
46:	HIV status
47:	HIV status
48:	HIV status
49:	Labour problems
50:	Labour problems
51:	Labour problems
52:	Labour problems
53:	Labour problems

```
54: Labour problems
55: Labour problems
56: Labour problems
57:             APH
58:             APH
59:             APH
60:             APH
61:             APH
62:             APH
63:             APH
64:             APH
65:    Hypertensive
66:    Hypertensive
67:    Hypertensive
68:    Hypertensive
69:    Hypertensive
70:    Hypertensive
71:    Hypertensive
72:    Hypertensive
         eff_mod
```
